# The *Drosophila* Chromodomain Protein Kismet Activates Steroid Hormone Receptor Transcription to Govern Axon Pruning and Memory *In Vivo*

**DOI:** 10.1016/j.isci.2019.05.021

**Published:** 2019-05-16

**Authors:** Nina K. Latcheva, Jennifer M. Viveiros, Daniel R. Marenda

**Affiliations:** 1 Department of Biology, Drexel University, 3141 Chestnut St., Philadelphia, PA 19104, USA; 2 Program in Molecular and Cellular Biology and Genetics, Drexel University College of Medicine, Philadelphia, PA, USA; 3 Department of Neurobiology and Anatomy, Drexel University College of Medicine, Philadelphia, PA, USA

**Keywords:** Neuroscience, Molecular Neuroscience, Cellular Neuroscience, Model Organism

## Abstract

Axon pruning is critical for sculpting precise neural circuits. Although axon pruning has been described in the literature for decades, relatively little is known about the molecular and cellular mechanisms that govern axon pruning *in vivo*. Here, we show that the epigenetic reader Kismet (Kis) is required for developmental axon pruning in *Drosophila* mushroom bodies. Kis binds to cis-regulatory elements of the steroid hormone receptor *ecdysone receptor* (*ecr*) gene and is necessary for activating expression of EcR-B1. Kis promotes the active H3K36 di- and tri-methylation and H4K16 acetylation histone marks at the *ecr* locus. We show that transgenic EcR-B1 can rescue axon pruning and memory defects associated with loss of Kis and that the histone deacetylase inhibitor SAHA also rescues these phenotypes. EcR protein abundance is the cell-autonomous, rate-limiting step required to initiate axon pruning in *Drosophila*, and our data suggest this step is under the epigenetic control of Kis.

## Highlights

• Kismet activates *ecr-b1* transcription to initiate developmental axon pruning
• Kismet promotes H3K36 di- and tri-methylation and H4K16 acetylation
• Axon pruning and memory defects of Kismet mutants are rescued by HDAC inhibition

## Graphical Abstract

**Figure:**
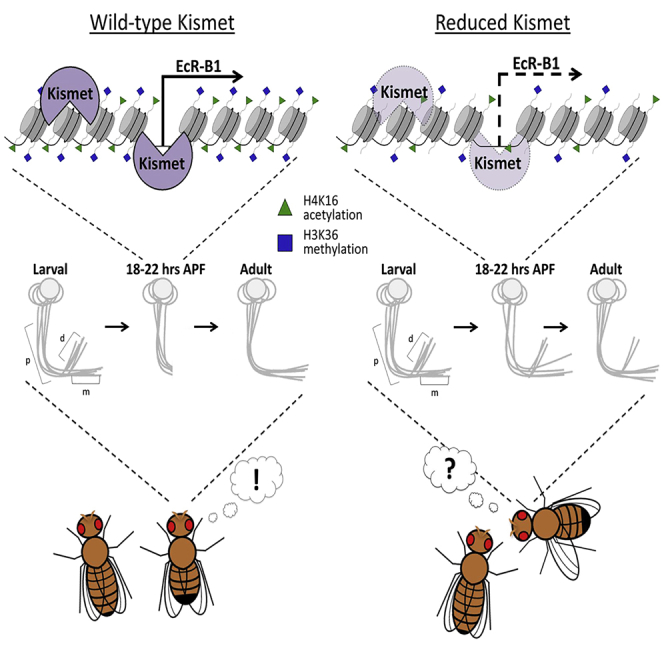

## Introduction

The elimination and refinement of synaptic connections is an integral part of normal development in vertebrates and invertebrates alike. Early in the developing nervous system, periods of progressive growth result in an overelaboration of synaptic connections onto a target. Inappropriate synapses then need to be eliminated to establish functional organization of the neuronal circuitry (Tau and Peterson, 2010). The pruning of these exuberant connections can occur on a small scale, as with dendritic remodeling, or on a large scale, such as with axon retraction and degeneration, with each type occurring through distinct molecular mechanisms (Low and Cheng, 2006). Precise control of axon pruning is critical for proper nervous system function, as defects in pruning have been well documented to lead to developmental neurological and psychiatric disorders (Tau and Peterson, 2010). Despite its vital role, relatively little is known about the mechanisms that govern axon pruning *in vivo*. What is well known about this process is that it requires tight regulation of gene expression to execute the necessary signaling pathways in a temporal and tissue-specific manner (Awasaki et al., 2011, Yu and Schuldiner, 2014). Epigenetic regulation is key to orchestrating precise gene expression programs for many tightly controlled processes in the body. However, its involvement in axon pruning is still unclear.

Holometabolous insects provide an attractive model for studying axon pruning as their nervous system undergoes extensive reorganization during metamorphosis (Levine et al., 1995, Truman, 1990). In *Drosophila melanogaster*, the larval neuronal circuitry is eliminated to make way for adult-specific circuitry that governs adult-specific behaviors. The most notable changes occur in the learning and memory processing center of the fly brain known as the mushroom bodies (MBs) (Connolly et al., 1996, Ferveur et al., 1995, Heisenberg et al., 1985, McBride et al., 1999). The MBs are bilaterally symmetrical structures in the central brain that are composed of ∼2,500 Kenyon cells divided into five populations of neurons: gamma, alpha, beta, alpha prime, and beta prime (Ito et al., 1997, Lee et al., 1999). Each Kenyon cell has dendrites, which extend into a structure called the calyx, as well as densely packed axons that make up the MB peduncle. From the peduncle, the axons then divide to form two separate lobes that extend into the dorsal and medial direction. The gamma neurons are generated first in development and initially during the larval stages extend bifurcated axons in the dorsal and medial lobes (Ito et al., 1997, Lee et al., 1999, Spindler and Hartenstein, 2010, Technau and Heisenberg, 1982). During metamorphosis, however, the gamma neuron axons are selectively pruned back to the peduncle to eliminate the bifurcation. At approximately 18–22 h after puparium formation (APF), the gamma neuron axons begin to re-extend new axons only into the medial lobe. This stereotypical developmental pruning of the gamma neurons has been shown to be initiated by the steroid hormone 20-hydroxyecdysone (ecdysone) (Awasaki et al., 2011, Lee et al., 2000, Zheng et al., 2003).

Ecdysone is most well known as the major molting hormone for its role in initiating each of the developmental transitions in arthropods (Handler, 1982). In *Drosophila*, ecdysone is released in large quantities by the prothoracic gland before each of the larval molts and pupation. The ligand is then able to enter the cytoplasm of target cells where it can bind to the Ecdysone Receptor (EcR). The binding of ecdysone to EcR stabilizes its interaction with its dimerization partner Ultraspiracle (Thummel, 2002, Yamanaka et al., 2013). The stable heterodimer enters the nucleus and activates transcription of a small subset of regulatory target genes known as immediate-early genes, which possess ecdysone response elements in the promotor regions (Ashburner, 1974, Ashburner et al., 1974, Thummel, 2002, Yamanaka et al., 2013). The specific responses different tissues have to induction of the ecdysone signaling cascade can be correlated to the three different EcR isoforms expressed in *Drosophila*: EcR-A, EcR-B1, and EcR-B2 (Talbot et al., 1993, Truman, 1990, Truman et al., 1994). The gamma neurons of the MBs in particular express EcR-B1, which has been shown to be a rate-limiting and cell-autonomous step required for the developmental pruning of axons during metamorphosis (Lee et al., 2000). In addition, EcR-B1 and functional gamma neurons in adult flies were shown to be required for short-term memory and the formation of courtship-associated long-term memory (Boulanger and Dura, 2015, Ishimoto et al., 2009, Redt-Clouet et al., 2012).

Our laboratory previously identified the chromodomain protein Kismet (Kis) as necessary for proper developmental axon pruning in the *Drosophila* MB neurons, although the mechanism by which Kis accomplished this was unknown (Melicharek et al., 2010). Kis is the *Drosophila* ortholog of the mammalian chromatin ATPase chromodomain helicase DNA-binding protein 7 (CHD7), a chromatin “reader” that is thought to play a role in chromatin remodeling by binding to methylated histone tails (Layman et al., 2010). In humans, heterozygous mutations in *CHD7* cause CHARGE syndrome (Vissers et al., 2004), an autosomal dominant neurodevelopmental disorder.

Here, we investigated the role of Kis in the developmental axon pruning of the *Drosophila* MB neurons. We determined that the loss of Kis in the MBs results in pruning defects during metamorphosis, which persist into adulthood and are due to a decrease in expression of *ecr-b1*. We show that endogenous Kis is enriched at a previously identified region of the genome shown to be important for *ecr* gene expression and that Kis binds to and is required to promote transcription from at least one cis-regulatory enhancer site in MB neurons. Furthermore, loss of Kis leads to a decrease in the histone marks H3K36 di- and tri-methylation (H3K36me2 and H3K36me3, respectively), which have been associated with actively transcribed genes in flies. Additionally, loss of Kis results in a striking loss of H4K16 acetylation (H4K16ac). Adult flies with Kis specifically decreased in the MB neurons display a loss of immediate recall memory, which is rescued by transgenic expression of EcR-B1. Finally, we show that pharmacological intervention via the general histone deacetylase (HDAC) inhibitor suberoylanilide hydroxamic acid (SAHA) can rescue the decrease in *ecr-b1* mRNA, axon pruning, and memory defects associated with decreased Kis in MB neurons. Taken together, these data show that Kis-mediated regulation of *ecr-b1* is required for proper developmental axon pruning *in vivo* by mediating the epigenetic marks H3K36me2, H3K36me3, and H4K16ac. These findings suggest that the rate-limiting step required to initiate axon pruning (*ecr-b1* expression) is under the epigenetic control of Kis.

## Results

### Kismet Is Required for MB Pruning

**Figure 1:**
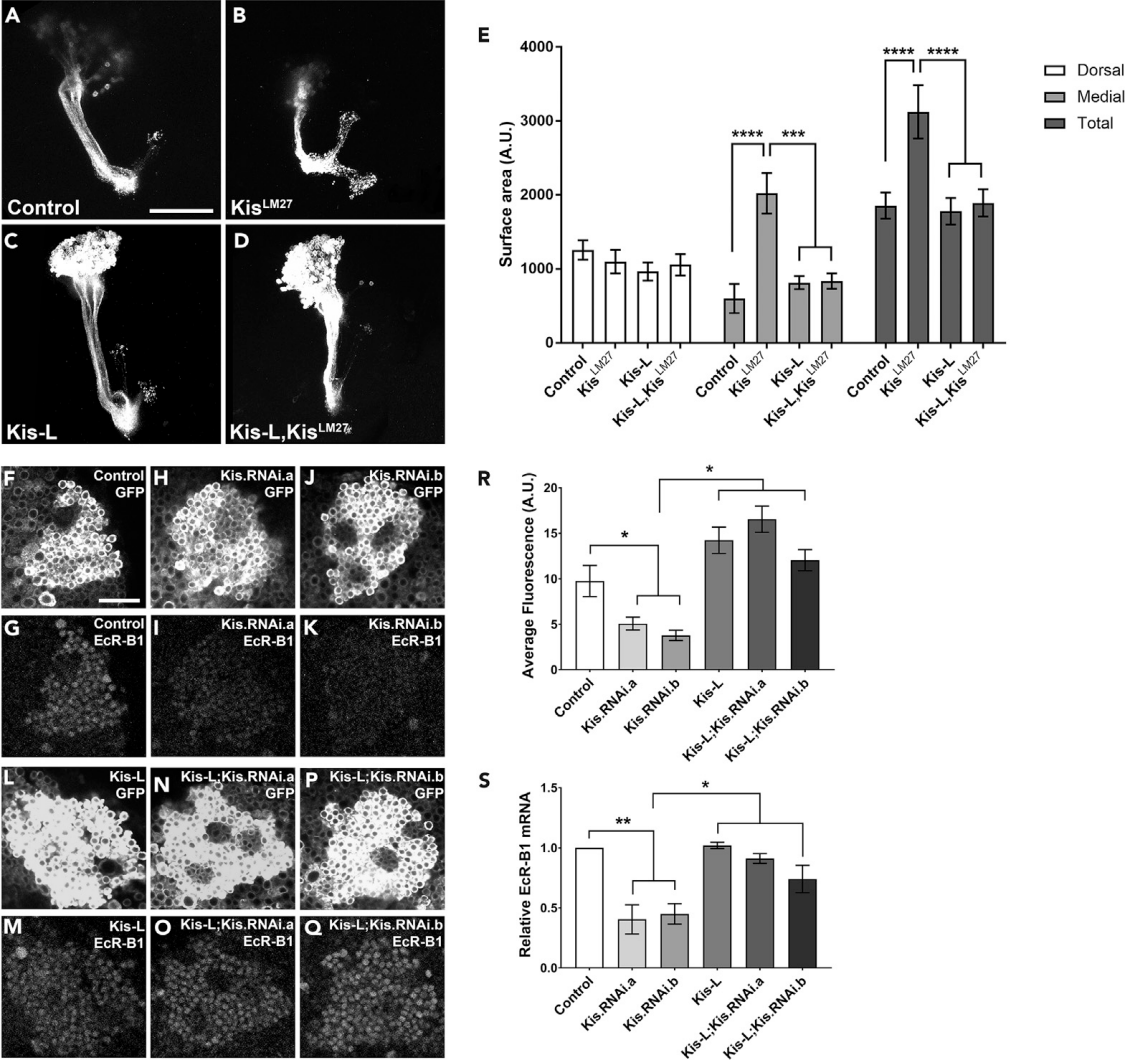
Kis Is Required for Developmental Axon Pruning and EcR Expression (A–D) Representative images of MARCM-generated MB clones expressing membrane-bound GFP using the *201y-Gal4* driver 18–22 h APF. (A) control (w^1118^; FRT40A), (B) kis null mutant (*Kis^LM27^,FRT 40A)*, (C) Kis overexpression (*UAS:Kis-L,FRT40A*), and (D) Kis rescue (*UAS:Kis-L,Kis^LM27^,FRT40A*). (E) Quantification of dorsal, medial, and total MB lobe surface areas in MARCM animals (from left to right, n = 12, 11, 10, 12 MBs). (F–Q) Representative images of pupal Kenyon cells using the *elav-Gal4,UAS:mCD8-GFP* driver 18–22 h APF. (F) control (*w*^*1118*^) membrane bound GFP, (G) control (*w^1118^*) EcR-B1 protein staining, Kis knockdowns: (H) GFP and (I) EcR-B1 in *UAS*:*Kis*.*RNAi*.*a* and (J) GFP and (K) EcR-B1 in *UAS*:*Kis*.*RNAi*.*b*. (L) Kis overexpression (*UAS*:*Kis-L*) GFP, and (M) EcR-B1 protein staining and Kis rescues: (N) GFP and (O) EcR-B1 in *UAS*:*Kis-L*; *UAS*:*Kis*.*RNAi*.*a,* and (P) GFP and (Q) EcR-B1 in *UAS*:*Kis-L*,*UAS*:*Kis*.*RNAi*.*b*. (R) Quantification of α-EcR-B1 fluorescence intensity within pupal Kenyon cells (from left to right, n = 10, 10, 10, 10, 7, 10 MBs). (S) Abundance of *ecr-b1* mRNA isolated from pupal heads analyzed by RT-qPCR using the *elav-Gal4* driver (number of biological replicates from left to right, 3, 3, 4, 3, 3, 3; 10 heads/biological replicate). Scale bars: 10 μm in (A) and 20 μm in (F). Statistical significance is represented by * = p < 0.05, ** = p < 0.01, *** = p < 0.001, and **** = p < 0.0001. Error bars represent the SEM.

We have previously shown that Kismet protein is widely expressed throughout the larval brain, including in the MB neurons (Melicharek et al., 2010). To characterize the pruning defects previously observed in *kis* mutant MB neurons, we utilized the mosaic analysis with a repressible cell marker (MARCM) system to generate homozygous mutant neuroblast clones tagged with a membrane-bound GFP (*UAS*:*mCD8-GFP*) using the *201y-Gal4* driver (Lee and Luo, 1999, Melicharek et al., 2010, Schuldiner et al., 2008, Yang et al., 1995). To quantify the pruning defects, we measured dorsal, medial, and total surface area of the MB lobes in pupal brains 18–22 h APF. This developmental window is standard in the field and has been extensively used because of the stereotypical timing in which axon pruning occurs in this model (Boulanger et al., 2011, Lai et al., 2016, Lee et al., 1999, Lee et al., 2000). At this time point, the MB lobes are mostly eliminated in control animals leaving only the peduncle (Figures 1A and 1E). In agreement with our previous work, the MB clones of the null mutant *kis*^*LM27*^ (Melicharek et al., 2008), MB clones had significantly larger medial and total lobe surface areas compared with control MB clones, indicative of unpruned axons (Figures 1A, 1B, and 1E) (Melicharek et al., 2010).

To expand upon our previous results, and to verify that loss of Kis was responsible for the MB pruning defect we observed, we expressed the wild-type Kis-L protein isoform (full length) in *kis*^*LM27*^ mutant MB clones. MB clones expressing Kis-L in the *kis*^*LM27*^ mutant background showed a significant reduction of the medial and total lobe areas (Figures 1D and 1E), suggesting the pruning defect is in fact due to loss of Kis protein function. Overexpression of Kis-L alone did not have an effect on lobe surface area compared with control MBs (Figures 1C and 1E). To verify the pruning defects we observed with the MARCM analysis, we also utilized a second knockdown system: RNA interference (RNAi)-mediated knockdown of Kis with two separate previously validated RNAi constructs (Melicharek et al., 2010). Expression of Kis-L using the neuronal *elav-Gal4* driver produces nearly 3-fold the normal amount of *kis* mRNA (Figure S1). Additionally, expression of *Kis-L* in conjunction with either *Kis*.*RNAi*.*a* or *Kis*.*RNAi*.*b* significantly reduced *kis* mRNA levels compared with *Kis-L* alone (Figure S1), which would compensate (at least partially) the loss of Kismet knockdown provided by Kismet RNAi. Pan-neural knockdown of Kis using the *elav-Gal4*,*UAS*:*mCD8-GFP* driver showed a significant increase in the medial and total lobe surface areas compared with outcross controls (Figure S2). Similar to the MARCM analysis, pan-neural expression of Kis-L in the knockdown genetic backgrounds was able to significantly rescue the medial and total surface area levels (Figure S2). Taken together, these data validate and expand upon our previous findings and show that Kis is required for the developmental axon pruning of MB gamma neurons during metamorphosis.

### Kismet Promotes EcR-B1 Protein and mRNA Expression

Expression of the steroid hormone receptor EcR-B1 is the first step in the developmental axon pruning of the MB neurons, and loss of EcR-B1 function produces defects in pruning similar to what we observe with decreased Kis function (Lee et al., 2000). Therefore, we sought to determine if Kis affects expression of EcR-B1. Pan-neural knockdown of Kis using the *elav-Gal4*,*UAS*:*mCD8-GFP* driver showed a significant decrease in EcR-B1 immunofluorescence in the MB Kenyon cells compared with those of control MBs at 18–22 h APF (Figures 1F–1K and 1R). Analysis of EcR-B1 protein staining during late third instar larval stage, when the ecdysone pulse is the highest, also reveals that MB Kenyon cells had a significant decrease of immunofluorescence levels upon loss of Kis (Figure S3) (Thummel, 2002). In support of these results, mRNA levels of *ecr-b1* were significantly reduced in pupal brains with pan-neural decreased expression of Kis as shown by RT-qPCR (Figure 1S). Additionally, replacement of Kis-L protein in the Kis knockdown background successfully rescued both decreased *ecr-b1* mRNA and EcR-B1 protein levels (Figures 1L–1S). Importantly, even though the *Kis-L* construct produces an overexpression of *kis* mRNA (Figure S1), this does not lead to a concomitant increase of EcR-B1 protein or mRNA levels outside of normal range (Figures 1R and 1S). This may be indicative of an upper limit to Kis's ability to promote EcR-B1 expression. Taken together, these data suggest that Kis is required to regulate EcR-B1 levels within MB neurons.

### Kismet Binds *Ecr* Locus *In Vivo*

**Figure 2:**
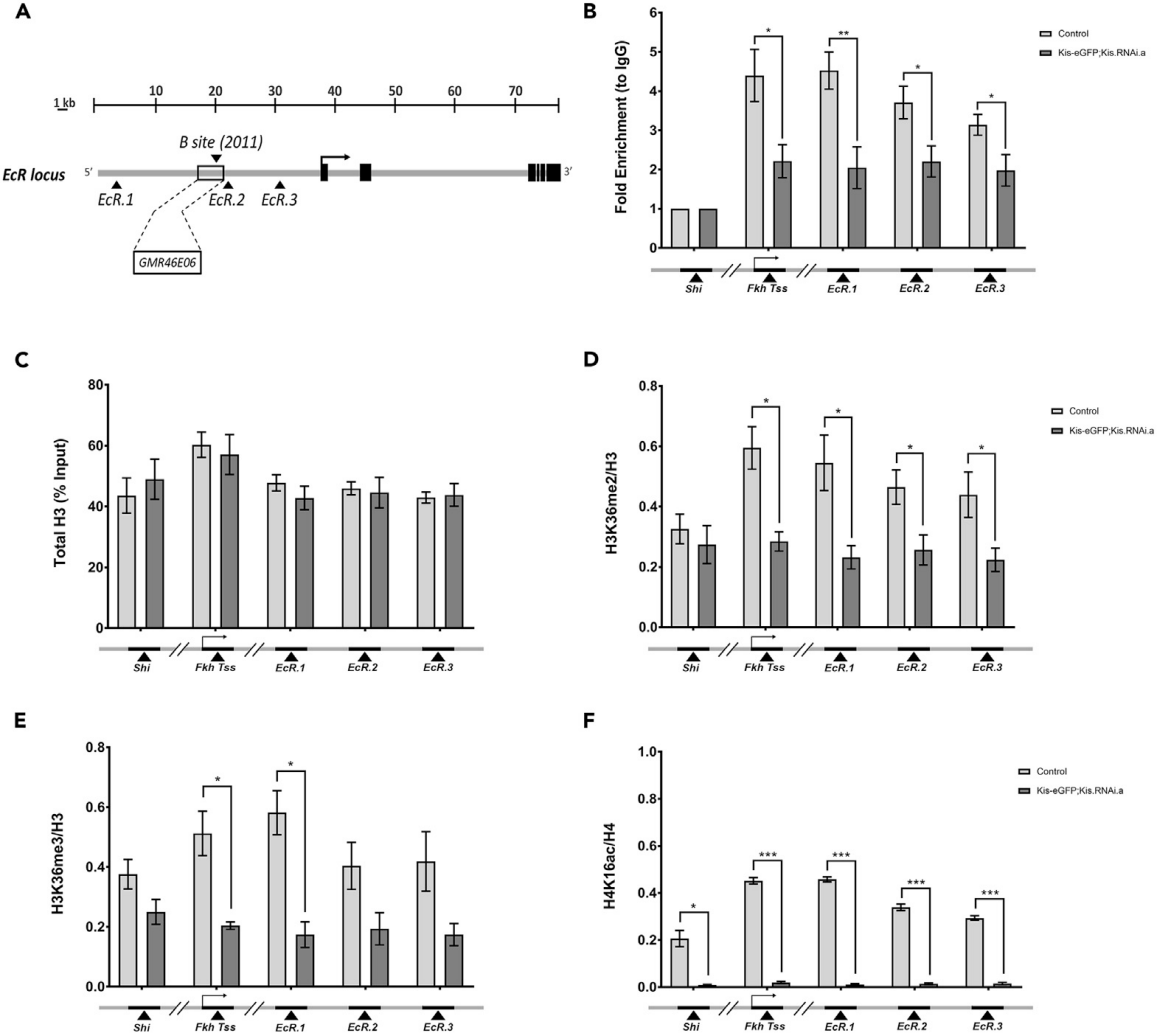
Kis Binds to *Ecr* Locus *In Vivo* Promoting H3K36 Methylation and H4K16 Acetylation (A) A schematic representation of the *ecr* locus. Black arrowheads upstream of the *ecr-b* TSS (black arrow) indicate location of primers. Black box denotes endogenous location of *GMR46E06-Gal4* enhancer site reporter. B set denotes location of primer set B from Boulanger et al. (Boulanger et al., 2011). (B) ChIP-qPCR analysis of chromatin isolated from third instar larval brains. Differences in Kis enrichment at the *ecr* enhancer sites (*EcR*.*1*, *EcR*.*2*, *and EcR*.*3*), the *fkh* TSS, and the *shi* promoter site between control (*Kis-eGFP*) and Kis knockdown (*elav-Gal4*; *Kis-eGFP/Kis-eGFP*; *Kis*.*RNAi*.*a/+*) animals displayed (n = 6 biological replicates). (C) Total H3 as a percentage of the input DNA was determined by qPCR at the previously noted genomic loci (from left to right, n = 5, 8, 8, 8, 8 biological replicates). (D and E) ChIP-qPCR analysis of (D) H3K36me2 and (E) H3K36me3 abundance relative to total H3 at the above-mentioned genomic loci, respectively (from left to right, n = 2, 4, 4, 4, 4 biological replicates). (F) ChIP-qPCR analysis of H4K16ac abundance relative to total H4 at the above-mentioned genomic loci (n = 3 biological replicates). Statistical significance is represented by * = p < 0.05, ** = p < 0.01, and *** = p < 0.001. Error bars represent the SEM.

Given that Kis is an epigenetic chromatin reader, we theorized that it may be affecting *ecr-b1* mRNA and EcR-B1 protein levels by promoting transcription. To begin testing this hypothesis, we performed chromatin immunoprecipitation (ChIP) followed by qPCR using chromatin isolated from third instar larval brains to analyze Kis occupancy at the *ecr* locus. We probed three cis-regulatory element sites between 7 and 33 kb upstream of the *ecr-b* transcription start site (TSS) (Figure 2A). We chose this genomic region for analysis as previous work showed that multiple transcription factor binding sites important for *ecr-b1* expression are present in this area (Figure 2A) (Boulanger et al., 2011). We utilized a Kis-eGFP protein trap animal previously described to express an enhanced Green Fluorescent Protein (eGFP) tagged version of the endogenous Kis protein (Buszczak et al., 2007, Ghosh et al., 2014). Importantly, the Kis-eGFP protein did not affect protein localization or function compared with wild-type Kis (Ghosh et al., 2014), and *UAS*:*Kis*.*RNAi*.*a* can successfully knock down the eGFP-tagged Kis protein and *kis-eGFP* mRNA (Figure S4). The *forkhead* (*fkh*) TSS served as a positive control, as it was previously reported to be bound by endogenous Kis (Srinivasan et al., 2008). Additionally, we used the *dynamin* homolog *shibire* (*shi*) as a negative control, since we had previously shown via microarray analysis that loss of Kis did not have any significant effect on *shi* mRNA expression (Ghosh et al., 2014). We verified that Kis was not enriched at the *shi* promoter region, and that knockdown of Kis did not have any significant effect on Kis abundance at the *shi* promoter (Figure 2B). In contrast, wild-type control brains showed enrichment of Kis at the three presumptive cis-regulatory sites in the *ecr* locus (*EcR*.*1*, *EcR*.*2*, *EcR*.*3*) (Figure 2B). Upon pan-neuronal knockdown of *Kis-eGFP*, we observed a significant decrease in enrichment at the *fkh* TSS, *EcR*.*1*, *EcR*.*2*, and *EcR*.*3* sites, confirming specificity for Kis binding (Figure 2B). These results suggest that Kis binds to presumptive cis-regulatory sites of the *ecr* locus in *Drosophila* third instar larval brains.

### Kismet Does Not Affect Nucleosomal Positioning at the *Ecr* Locus

One mechanism by which chromatin remodeling proteins promote transcriptional activation is by mobilizing nucleosomes and allowing transcriptional machinery access to enhancer sites and promoters of target genes (Clapier et al., 2017). Given Kis's homology to CHD7 and its conserved ATPase domain, we hypothesized that Kis may be remodeling nucleosomes at the *ecr* locus to allow access for the transcriptional machinery. To test this possibility, we immunoprecipitated total histone 3 (H3) protein at the *ecr* locus in control and Kis knockdown third instar larval brains. We found no significant difference between these conditions at each of the cis-regulatory loci we analyzed (Figure 2C). To verify this, we performed an MNase protection assay, which utilizes an endo-exonuclease to examine the nucleosomal occupancy at desired loci by quantifying the amount of DNA bound and thus protected by the nucleosomes (Berson et al., 2017). Consistent with our total H3 analysis, we observed no change in the quantity of DNA digested upon Kis knockdown (Figure S5). Taken together, these data suggest that Kis is not affecting the movement of nucleosomes at the *ecr* locus as a mechanism to promote expression of *ecr-b1*.

### Kismet Does Not Affect H3K4 or H3K27 Methylation at the *Ecr* Locus

Another way chromatin readers can affect gene expression is by altering the histone modifications present at relevant genomic loci (Clapier et al., 2017), and Kis has been shown to affect histone modifications previously (Srinivasan et al., 2008). We began by analyzing H3K4 methylation states (mono-, di-, and tri-methylation) at the *ecr* locus in control and Kis knockdown animals, as it is the type of methylation most commonly associated with actively transcribed genes. ChIP-qPCR analysis showed no change in all types of H3K4 methylation levels upon Kis knockdown at these loci (Figures S6A–S6C). We next analyzed H3K27 trimethylation (H3K27me3), as this modification is often associated with transcriptional repression and has been previously shown to be increased in *kis* mutant polytene salivary glands (Srinivasan et al., 2008). We did not observe any significant change in H3K27me3 upon Kis knockdown at any of the loci examined (Figure S6D). These data suggest that alteration of H3K4 methylation or H3K27 trimethylation is not part of the mechanism by which Kis controls *ecr* gene expression in the *Drosophila* larval CNS.

### Kismet Promotes H3K36 Methylation and H4K16 Acetylation

Previous studies demonstrated a global decrease in H3K36me2 and H3K36me3 upon Kis loss in *Drosophila* larval salivary gland polytene chromosomes (Dorighi and Tamkun, 2013). This type of modification is usually associated with actively transcribed genes in *Drosophila* (Dorighi and Tamkun, 2013, Stabell et al., 2007, Wagner and Carpenter, 2012). We therefore sought to determine if the same effect on H3K36 methylation was present in the *Drosophila* larval nervous system upon pan-neural knockdown of Kis protein. We observed that H3K36me2 was significantly decreased at all of the putative *ecr* cis-regulatory sites we analyzed, as well as at the *fkh* positive control, in Kis knockdown brains compared with controls (Figure 2D). Additionally, H3K36me3 was also significantly decreased at *EcR*.*3* and the *fkh* TSS (Figure 2E). Importantly, this decrease in H3K36 methylation was not global, as there was no significant change with either H3K36 di- or tri-methylation at the *shi* promoter, which is not bound by Kis (Figures 2B, 2D, and 2E). Also, given that H3K36me2 is the substrate for the tri-methylated form, it is likely that the decrease in H3K36me3 is due to the decrease in H3K36me2. Combined, these results suggest Kis affects H3K36me2 and H3K36me3 histone marks at putative cis-regulatory elements upstream of the *ecr* TSS in third instar larval brains.

Previous studies with H3K36me2 have demonstrated a synergistic relationship with other histone modifications, particularly H4K16ac (Bell et al., 2007). H4K16ac directly influences transcription by positively regulating chromatin accessibility to non-histone proteins (Zhang et al., 2017). Additionally, this type of post-translational modification has been shown to negatively impact chromatin condensation by preventing the function of ATP-dependent chromatin-assembly factor, which is involved in the condensation of 30-nm chromatin fibers (Shogren-Knaak et al., 2006, Yang et al., 2006). Since we saw a significant decrease in H3K36me2 at our loci of interest, we wanted to determine if H4K16ac levels were also affected in Kis knockdown animals. ChIP-qPCR revealed that H4K16ac was significantly decreased when Kis was knocked down compared with controls (Figure 2F). This was consistent at all of the loci analyzed, including the *shi* promoter, possibly indicative of a universal decrease in H4K16ac. These results suggest a role for Kismet in maintaining the active histone modifications H3K36me2, H3K36me3, and H4K16ac at cis-regulatory sites as a potential mechanism for activating gene expression.

### Kismet Promotes Transcription *In Vivo*

**Figure 3:**
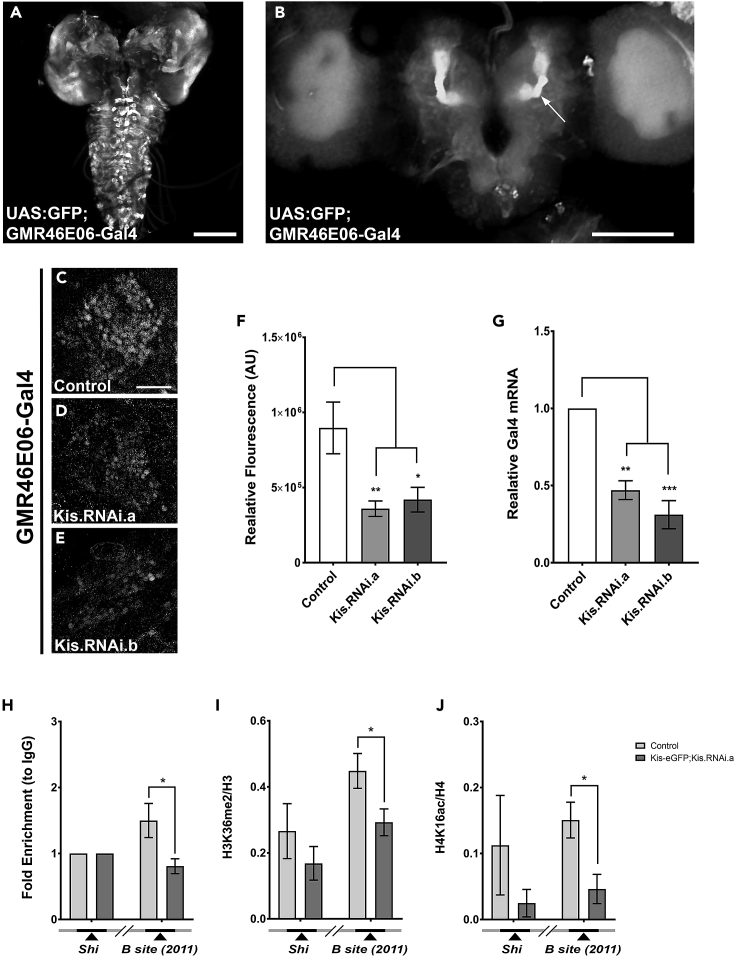
Kis Can Promote Transcription via Putative Enhancer Site 16 kb Upstream of EcR-B TSS (A and B) Representative images of UAS:GFP; GMR46E06-Gal4 expression in larval and pupal brain, respectively. Arrow in (B) denotes location of MB peduncle area. (C–E) Representative images of (C) control (*w*^*1118*^) and Kis knockdowns (D) (*UAS*:*Kis*.*RNAi*.*a*) and (E) (*UAS*:*Kis*.*RNAi*.*b*) in larval MB Kenyon cells stained with α-Gal4 expressed using the *GMR46E06-Gal4* enhancer site reporter. (F) Quantification of fluorescent intensity of α-Gal4 (from left to right, n = 10, 10, 8 MBs). (G) *gal4* mRNA levels analyzed by RT-qPCR (n = 3, 10 heads/biological replicate). (H–J) ChIP-qPCR analysis of (H) Kis enrichment, (I) H3K36me2, and (J) H4K16ac at B site 2011 from Boulanger et al., (2011), respectively (from left to right, n = 5, 5, 2, 3, 6, 7 biological replicates). Scale bars: 4 μm in (A), 100 μm in (B), and 20 μm in (C). Statistical significance is represented by * = p < 0.05, ** = p < 0.01, and *** = p < 0.001. Error bars represent the SEM.

To determine if Kis can specifically control transcriptional output from this genomic area, we utilized a transcriptional reporter that was generated as part of an effort to find putative brain enhancers in *Drosophila* (*GMR46E06-Gal4*, described in Pfeiffer et al., 2008). This reporter drives the expression of transgenic Gal4 protein from a 3,999-kb region endogenously located 16 kb upstream of the *ecr-b* TSS. The 3,999-kb region contains two binding sites of the transcription factor FTZ-F1, which has previously been shown to be required for *ecr* gene expression (Boulanger et al., 2011), and is capable of driving expression of Gal4 in larval, pupal, and adult MBs (Figures 3A and 3B), suggesting this is a *bona fide* enhancer region for *ecr* transcription and underscoring the importance of *GMR46E06* as a CNS enhancer. We hypothesized that, if Kis is required for promoting transcriptional output from this region *in vivo*, then expressing the *UAS*:*Kis*.*RNAi* in the *GMR46E06-Gal4* background will decrease Kis protein levels and in turn decrease Gal4 transcriptional output. We therefore measured the levels of *gal4* mRNA and Gal4 protein in control and Kis knockdown brains via RT-qPCR and in MB neurons specifically via immunohistochemistry (Figures 3C–3G). We observed a significant decrease in both *gal4* mRNA and Gal4 protein levels from this reporter in Kis knockdown animals compared with controls, suggesting Kis is required for promoting transcriptional output from this *GMR46E06* region *in vivo* (Figures 3C–3G).

We next wanted to determine if Kis is enriched at this region by performing ChIP-qPCR. We utilized a previously validated site (B site [2011], Figure 2A) within the *GMR46E06* region to test for binding as this site was also bound by the FTZ-F1 transcription factor, which is required for *ecr-b1* gene expression (Boulanger et al., 2011). We found that Kis is enriched at this site and that RNAi-mediated knockdown of Kis-eGFP significantly decreased this enrichment (Figure 3H). We next sought to determine if Kis is also affecting H3K36me2 and H4K16ac modifications at the *GMR46E06* site as it does at the other loci we have tested. We found that loss of Kis significantly decreased both H3K36me2 and H4K16ac (Figures 3I and 3J). Additionally, there was no significant difference in the total histone 3 levels at this site upon Kis knockdown (Figure S7), consistent with our findings at the other loci we analyzed. Taken together, these data suggest that Kis protein can promote transcriptional activation, and potentially increase EcR-B1 expression, by increasing H3K36me2 and H4K16ac in the *Drosophila* nervous system. Additionally, the Kismet homolog (CHD7) has been suggested to bind to sites distal to TSSs, sites with features suggesting that they are gene enhancer elements. Thus, these data are consistent with at least one other CHD protein binding function (Schnetz et al., 2009).

### EcR-B1 Rescues Kis Loss of Function Axon Pruning Defects

**Figure 4:**
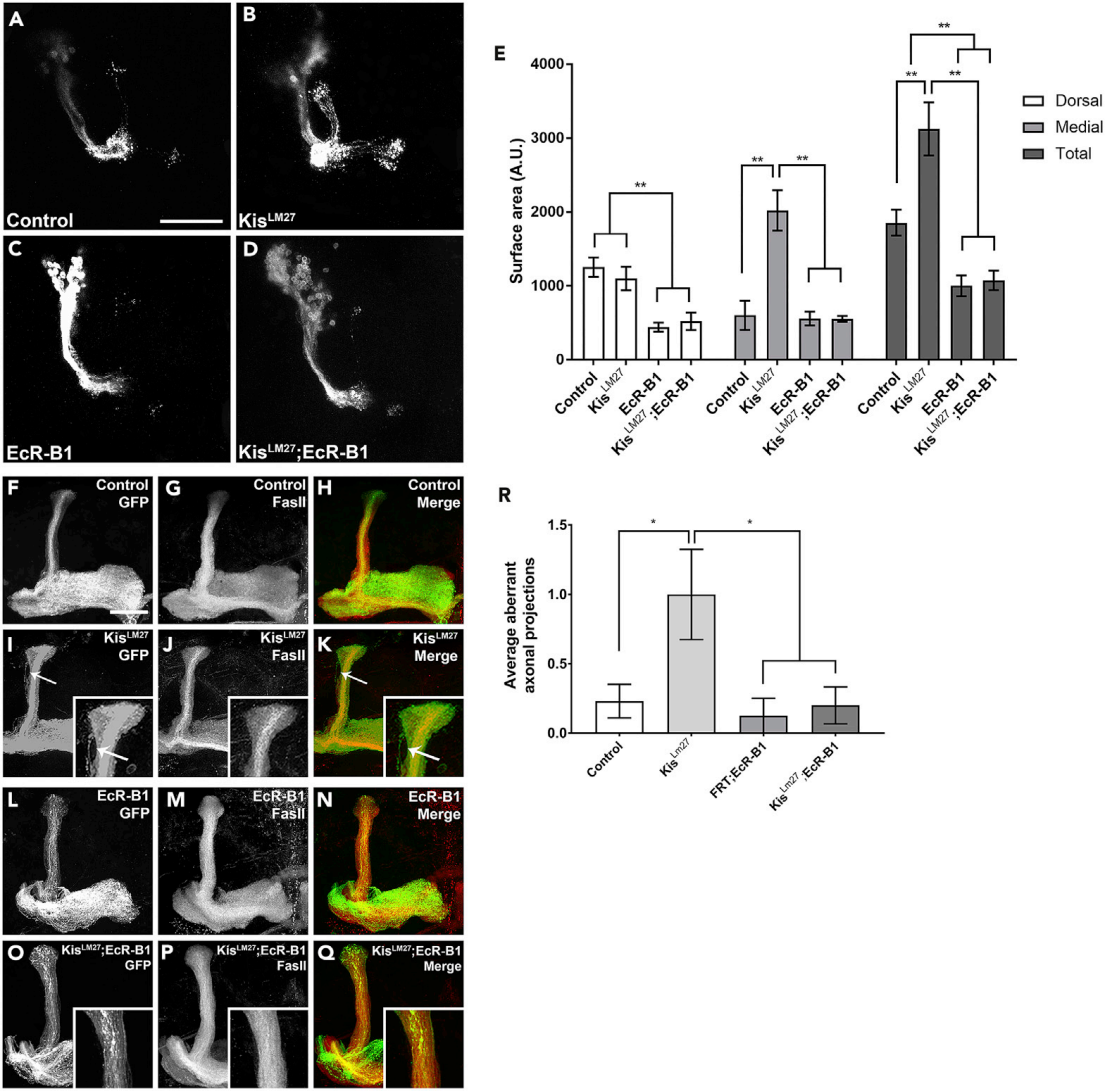
Transgenic EcR-B1 Rescues Defective Axon Pruning Associated with Loss of Kis (A–D) Representative images of MARCM-generated MB clones expressing membrane-bound GFP using the *201y-Gal4* driver 18–22 h APF. (A) control (*w^1118^; FRT40A)*, (B) *kis* null mutant (*Kis^LM27^,FRT40A)*, (C) EcR-B1 overexpression (*FRT40A; UAS:EcR-B1*), and (D) EcR-B1 rescue (*Kis^LM27^,FRT40A; UAS:EcR-B1*). (E) Quantification of dorsal, medial, and total MB lobe surface areas in MARCM animals (from left to right, n = 12, 11, 10, 12 MBs). (F–Q) Representative adult MARCM-generated MB clones expressing membrane-bound GFP or stained with α-FasII. (F) control (*w^1118^; FRT40A)* membrane-bound GFP, (G) α-FasII immunostaining, and (H) merge images. (I) *kis* null mutant (*Kis^LM27^,FRT40A)* GFP, (J) α-FasII, and (K) merge. (L) EcR-B1 overexpression alone (*FRT40A; UAS:EcR-B1*) GFP, (M) α-FasII immunostaining, and (N) merge. (O) EcR-B1 rescue (*Kis^LM27^,FRT40A; UAS:EcR-B1*) GFP, (P) α-FasII immunostaining, and (Q) merge. Arrows indicate aberrant axonal projections in (I) and (K). Insets show magnified area of medial lobes. (R) Quantification of average aberrant axonal projections in MBs (from left to right, n = 13, 12, 8, 10 MBs). Scale bars: 10 μm in (A) and 20 μm in (F). Statistical significance is represented by * = p < 0.05, ** = p < 0.01, and *** = p < 0.001. Error bars represent the SEM.

EcR-B1 is well documented to be a key player in initiating the axon pruning cascade in MB neurons (Lee et al., 2000, Yu and Schuldiner, 2014). Given that we have shown Kis acts to promote expression of *ecr-b1* in MB neurons from at least one transcriptional enhancer, we sought to determine if we could rescue the pruning defects we observe in *kis* mutants by expressing transgenic EcR-B1. Utilizing the MARCM system, we expressed transgenic EcR-B1 protein within *kis*^*LM27*^ mutant MB clones (Figure 4). We observed that transgenic expression of EcR-B1 significantly reduced the abnormal pruning observed in *kis*^*LM27*^ mutant MB clones (Figures 4B, 4D, and 4E). We also observed a significant reduction in pruning defects when transgenic EcR-B1 was pan-neurally co-expressed with *Kis*.*RNAi* constructs using the *elav-Gal4*,*UAS*:*mCD8-GFP* driver (Figure S8). Interestingly, EcR-B1 overexpression alone, as well as EcR-B1 expression in Kis loss of function backgrounds, produced smaller surface areas in the dorsal and total lobes compared with outcross controls (Figures 4A, 4C, and 4E). This is consistent with Kis functioning upstream of EcR-B1 in the pruning process, as well as with EcR-B1 being the rate-limiting factor for pruning. Therefore, these data suggest that Kis mediates axon pruning in pupal MB neurons by transcriptionally activating *ecr-b1*, thereby controlling EcR-B1 protein levels.

Pruning defects observed during metamorphosis may simply reflect a delay in normal pruning. Therefore, to determine that homozygous MARCM *kis*^*LM27*^ mutant clones indeed have defective developmental pruning, as opposed to having delayed pruning, we sought to determine if the unpruned axons persisted into adulthood. We generated MARCM clones with homozygous *kis*^*LM27*^ MBs (as previously described) and aged the adults for 5 days after eclosion. At the adult stage, the *201y-Gal4* driver is expressed in a subset of alpha and beta neurons in addition to all the gamma neurons (Bornstein et al., 2015). To differentiate, with certainty, between novel pupal-stage generated axons and pre-pupal stage retained axons, we chose to examine an area that should not contain many GFP-positive bundles, i.e., the dorsal lobe. We then immunostained the adult brains with anti-FASII, a transmembrane cell adhesion protein, which is differentially expressed in the separate populations of MB neurons (Bornstein et al., 2015, Stewart and McLean, 2004). FASII expression is lowest in the early born gamma neurons and highest in the late born alpha/beta neurons in adult MBs; therefore, the appearance of GFP-labeled MARCM axons in the dorsal lobe that are weakly or unstained for FASII would constitute aberrant unpruned axons that persisted into adulthood (Bornstein et al., 2015). Compared with control MBs, *kis*^*LM27*^ MARCM clones had significantly more weakly-stained and/or unstained FASII GFP-positive axons outside the dorsal lobe bundle, indicating that pruning is in fact prevented and not delayed in this mutant (Figures 4F–4K and 4R). Given that transgenic expression of EcR-B1 in the *kis*^*LM27*^ MARCM background rescued the pruning defects during the pupal stage, we tested whether transgenic EcR-B1 can also rescue the presence of aberrant axons in adulthood. Expression of EcR-B1 in *kis*^*LM27*^ mutant MARCM clones showed significantly fewer GFP-positive axons outside the dorsal lobe in the adult MB (Figures 4I, 4L–4Q, and 4R). Collectively, these data suggest that the defective pruning observed during metamorphosis in *kis* loss-of-function MB neurons persists into adulthood.

### EcR-B1 Rescues Memory Defects

**Figure 5:**
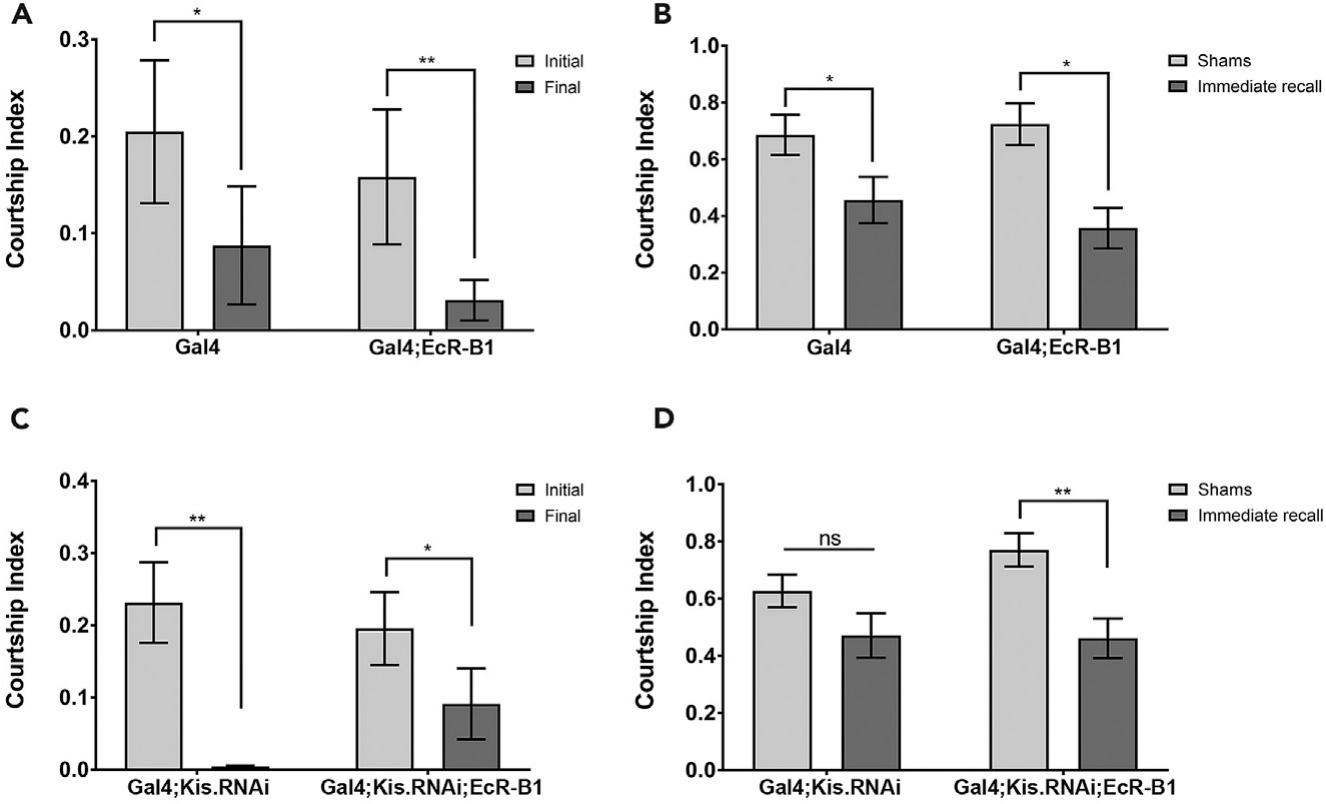
Transgenic EcR-B1 Expression Rescues Memory Defects Rescued in Kis Knockdown MBs (A and C) Assessment of male courtship index during the initial and final 10 min of the 60-min training period ([A] from left to right, n = 16, 15 males; [C] from left to right, n = 21, 22 males). (B and D) Immediate recall of trained males was assessed and compared with genetically identical sham trained males ([B] from left to right, n = 14, 13, 13, 15 males; [D] n = 21, 21, 19, 22 males). Statistical significance is represented by * = p < 0.05 and ** = p < 0.01. Error bars represent the SEM.

Previous studies from our laboratory showed that reduction of Kis levels in MB neurons produced significant defects in immediate recall memory (Melicharek et al., 2010). Next, we wanted to determine if transgenic expression of EcR-B1 could also rescue the memory defect associated with loss of Kis function. We utilized the conditioned courtship suppression assay, which takes advantage of the innate courting behaviors carried out by male *Drosophila* in response to multimodal signals transduced by females (McBride et al., 2005, Siegel and Hall, 1979, Siwicki et al., 2005). Wild-type males will decrease their rate of courting during a training (learning) period of one hour with an unresponsive female. These males will continue to court at lower rates even with subsequent receptive females for an average of 1–3 h after exposure (McBride et al., 2005, Siegel and Hall, 1979, Siwicki et al., 2005). We utilized *UAS*:*Kis*.*RNAi*.*a* to knock down Kis in MB neurons using the *ok107-Gal4* driver. Importantly, expression of the Gal4 alone or Gal4-mediated expression of transgenic EcR-B1 did not produce any learning (Figure 5A) or memory (Figure 5B) defects. We observed that males with decreased Kis protein displayed intact learning, as evident by the significant decrease in courtship from the initial to final stages of exposure to an unresponsive female (Figure 5C). Similarly, males with both decreased Kis and transgenic EcR-B1 also had intact learning (Figure 5C). However, males with decreased Kis showed abnormal memory (Figure 5D), as these males had rates of courtship that were not significantly different from those of sham males, which did not receive exposure to an unreceptive female. In contrast, trained males with both decreased Kis and transgenic EcR-B1 showed significantly reduced courtship compared with sham males, indicative of intact immediate recall memory (Figure 5D). Taken together, these data suggest that the memory defects associated with loss of Kis in the MB neurons are due to decreased EcR-B1 levels.

### SAHA Rescues Morphological and Behavioral Defects Associated with Loss of Kis

**Figure 6:**
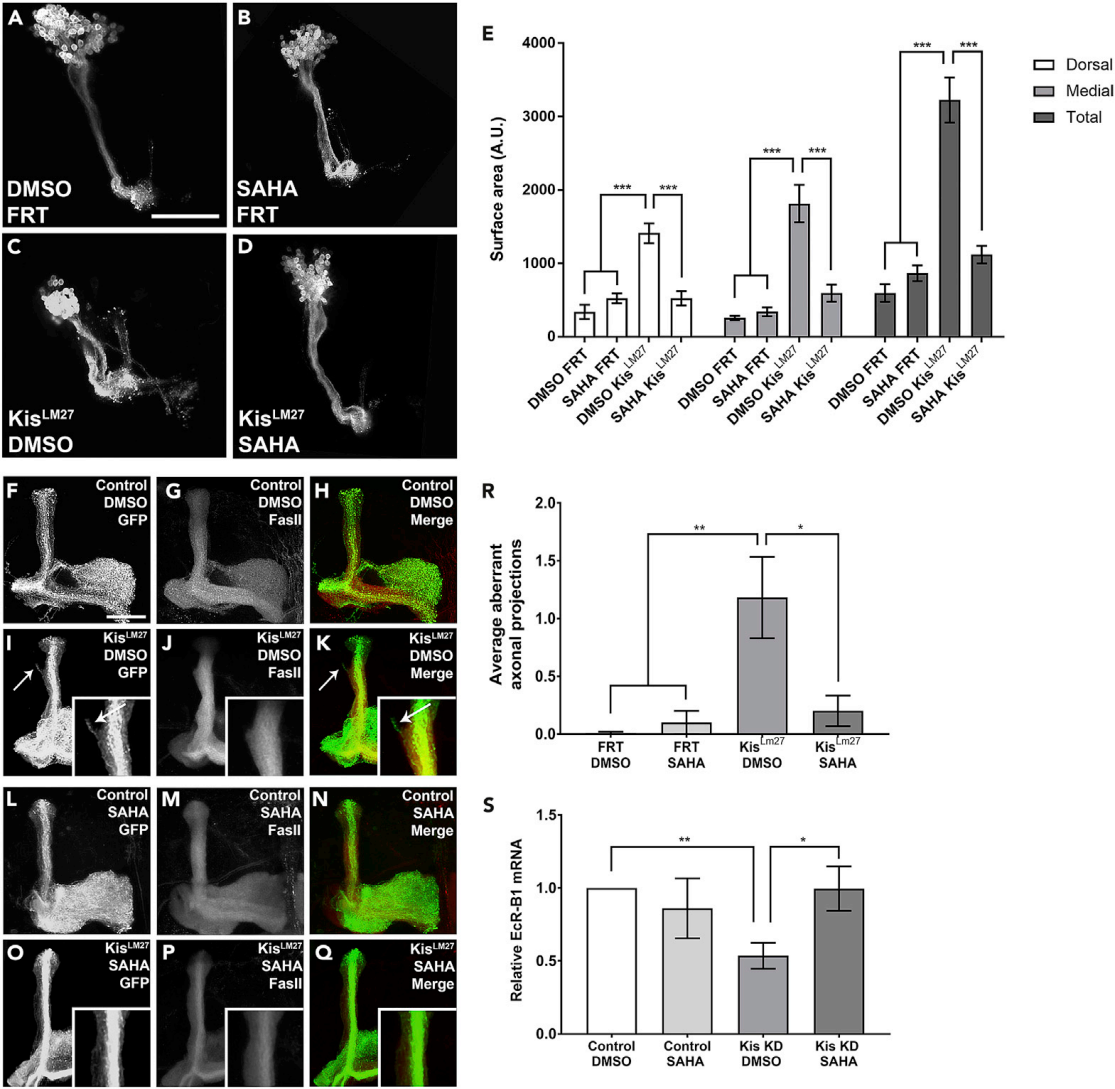
HDAC Inhibition Rescues Defective Axon Pruning and EcR-B1 Expression in Loss of Kis Animals (A–D) Representative images of DMSO- or SAHA-treated MARCM-generated MB clones expressing GFP using the *201y-Gal4* driver 18–22 h APF. (A) DMSO-treated control (*w^1118^; FRT40A)*, (B) SAHA-treated control (*w^1118^; FRT40A)*, (C) DMSO-treated *kis* null mutant (*Kis^LM27^, FRT40A)*, and (D) SAHA-treated *kis* null mutant (*Kis^LM27^,FRT40A).* (E) Quantification of dorsal, medial, and total MB lobe surface areas in DMSO- or SAHA-treated MARCM animals (from left to right, n = 11, 11, 11, 10 MBs). (F–Q) Representative DMSO- or SAHA-treated adult MARCM-generated MB clones expressing membrane-bound GFP or stained with α-FasII. (F) DMSO-treated control (*w^1118^; FRT40A)* membrane-bound GFP, (G) α-FasII immunostaining, and (H) merge images. (I) DMSO-treated *kis* null mutant (*Kis^LM27^, FRT40A)* GFP, (J) α-FasII, and (K) merge. (L) SAHA-treated control (*w^1118^; FRT40A)* GFP, (M) α-FasII immunostaining, and (N) merge. (O) SAHA-treated *kis* null mutant (*Kis^LM27^, FRT40A)* GFP, (P) α-FasII immunostaining, and (Q) merge. Arrows indicate aberrant axonal projections in (I) and (K). Insets show magnified area of medial lobes. (R) Quantification of average aberrant axonal projections in MBs of flies treated with DMSO or SAHA (from left to right, n = 10, 10, 11, 10 MBs). (S) *ecr-b1* mRNA levels isolated from DMSO- or SAHA-treated control (*w*^*1118*^) and pan-neural Kis knockdown (*UAS*:*Kis*.*RNAi*.*a*) pupal heads analyzed by RT-qPCR (number of biological replicates from left to right, n = 4, 3, 4, 4; 10 heads/biological replicate). Scale bars: 10 μm in (A) and 20 μm in (F). Statistical significance is represented by * = p < 0.05, ** = p < 0.01, and *** = p < 0.001. Error bars represent the SEM.

**Figure 7:**
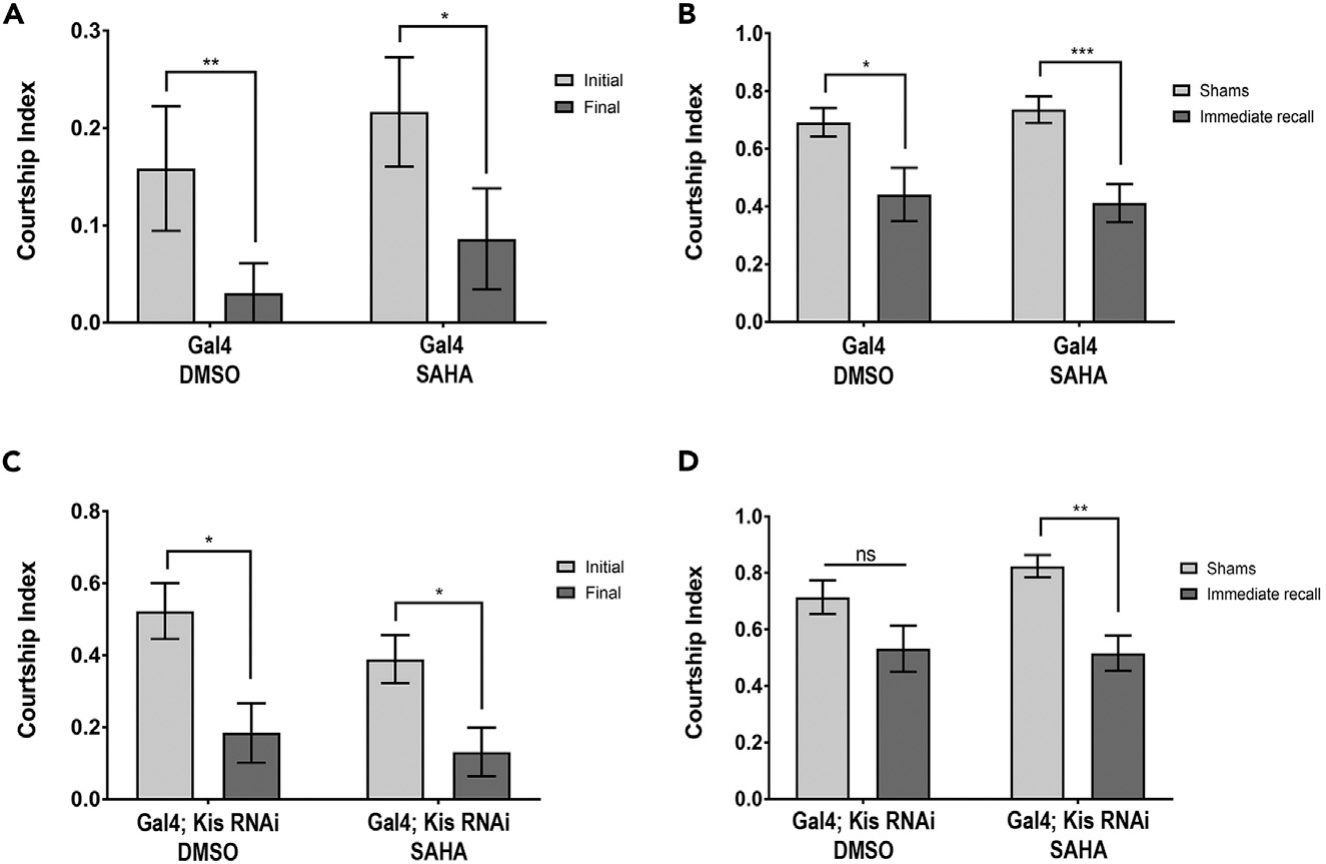
SAHA Treatment Rescues Immediate Recall Defects Rescued in Kis Knockdown Animals (A and C) Assessment of DMSO- or SAHA-treated male courtship index during the initial and final 10 min of the 60-min training period ([A] from left to right, n = 14, 23 males; [C] from left to right, n = 19, 22 males). (B and D) Immediate recall of DMSO- or SAHA-treated trained males assessed and compared with sham trained males with identical genetic background and treatment ([B] from left to right, n = 29, 15, 16, 23 males; [D] n = 22, 18, 21, 22 males). Statistical significance is represented by * = p < 0.05 and ** = p < 0.01. Error bars represent the SEM.

Recently, our laboratory showed that pharmacological inhibition of HDACs can rescue multiple defects associated with loss of Kis at the neuromuscular junction (Latcheva et al., 2018). HDAC inhibition (HDACi) did not significantly affect *kis* mRNA levels (Latcheva et al., 2018), so we hypothesized that the rescue may be due to effects on a common set of target genes. To further examine this hypothesis, we tested whether HDACi treatment could rescue the decreased EcR-B1 expression, pruning, and memory defects observed in Kis knockdown animals. We observed that treatment of Kis knockdown with SAHA significantly increased *ecr-b1* mRNA levels compared with DMSO-treated controls (Figure 6S). Importantly, SAHA treatment alone had no significant impact on *ecr-b1* mRNA levels (Figure 6S) or axon pruning in either pupal (Figures 6A, 6B, and 6E) or adult MBs (Figures 6F–6H, 6L–6N, and 6R). However, we did observe that SAHA treatment significantly decreased the number of unpruned axons in both pupal (Figures 6C, 6D, and 6E) and adult brains in *kis*^*LM27*^ mutant MB neurons (Figures 6I–6K, 6O–6Q, and 6R). Finally, SAHA treatment itself did not have an observable impact on learning or memory in controls (Figures 7A and 7B, respectively), or learning in Kis knockdown (Figure 7C). However, SAHA treatment was able to significantly rescue the immediate recall defect in Kis knockdown animals compared with DMSO treatment alone (Figure 7D). In each case, SAHA affected these phenotypes only in *kis* loss-of-function animals, and not in control animals, suggesting a specificity of SAHA interaction with Kis function. SAHA’s theraputic effect might be by counteracting the loss of H4K16 acetylation we observed in Kis knockdown animals. Taken together, these results show that HDACi treatment significantly rescues multiple defects associated with Kis loss of function.

## Discussion

Axon pruning and elimination are critical steps to establishing and refining neural circuitry. However, relatively little is known about how the precise extrinsic and intrinsic signals come together to initiate the pruning cascade. Here, we begin to unravel the epigenetic mechanisms essential for initiating developmental axon pruning *in vivo*. We show that the chromatin reader Kis activates transcription of *ecr-b1* in the *Drosophila* MBs and promotes methylation of H3K36 and acetylation of H4K16 at cis-regulatory sites of the *ecr-b1* locus. Proper regulation of *ecr-b1* expression by Kis is required for both pruning and immediate recall memory in adults. Finally, we show that the general HDACi, SAHA, can increase *ecr-b1* mRNA levels in animals with decreased Kis and rescue their pruning and memory defects. SAHA may be counteracting the loss of H4K16ac and reestablish a balance of gene expression in Kis knockdown animals. Taken together, our data show that the essential rate-limiting step in developmental axon pruning, EcR-B1 expression, is under the epigenetic control of Kis.

The expression of *ecr* in the MBs has been subject to studies indicating two distinct pathways of activation (Boulanger et al., 2011, Boulanger and Dura, 2015, Zheng et al., 2003). First, the dTGF-β signaling pathway has been implicated in MB gamma neuron remodeling, as mutations in both the *Drosophila* TGF-β receptor, Baboon, and its downstream effector, dSmad2, produce pruning defects at 18–22 h APF (Zheng et al., 2003). Furthermore, transgenic expression of EcR-B1 was able to rescue the pruning defects in the TGF-β pathway mutant backgrounds indicating that EcR-B1 is downstream of TGF-β signaling (Zheng et al., 2003). Similarly, our results demonstrate that transgenic EcR-B1 expression bypassed the need for Kis in a *kis* mutant background and thereby rescued the pruning defects in both pupal and adult MBs. This parallels the previous findings by demonstrating that Kis is upstream of *ecr-b1* transcription. Where Kis fits relative to dTGF-β signaling, however, still remains to be answered.

A second pathway of activating *ecr-b1* transcription in the MBs is via the nuclear receptor Ftz-f1 and its homologue Hr39 (Boulanger et al., 2011, Boulanger and Dura, 2015). Ftz-f1 binds directly to the *ecr* cis-regulatory sites and activates expression of *ecr-b1*, whereas Hr39 acts to block its expression in the MBs (Boulanger et al., 2011). Although this pathway also functions upstream of *ecr-b1* transcription, it is independent of dTGF-β signaling as Ftz-f1 overexpression did not rescue pruning defects in *baboon* mutants (Boulanger et al., 2011). Our data indicate that Kis is also enriched at one cis-regulatory site that Ftz-f1 binds (Figures 2A and 3H), but whether they act in coordination or independently of each other remains to be determined. It may be that both Kis and Ftz-f1 act independently and help create redundancy in case of the failure of one pathway. Given that reduction of neither Kis nor Ftz-f1 completely eliminated EcR-B1 levels may support this redundancy hypothesis; however, more work is necessary to explore their relationship (Boulanger et al., 2011).

Few studies have hinted at an epigenetic mechanism of *ecr* transcriptional activation. The *Drosophila* Set2 K36 histone methyl transferase (HMT) was shown to genetically interact with the EcR signaling pathway and positively regulate expression of EcR target genes (Stabell et al., 2007). However, as dSet2 is required for general transcriptional elongation, this may be a global effect of dSet2 function. Nonetheless, this implicates H3K36 methylation as an important factor in *ecr* expression. Although Kis does not appear to affect expression of *Drosophila set2*, and the other H3K36 HMT genes *ash1* or *dmes-4* (data not shown), it might be acting to recruit the HMTs to relevant target genes to transduce effects on transcriptional regulation. To this effect, Kis was previously shown to increase the global association of Ash1 on polytene chromosomes (Dorighi and Tamkun, 2013). Although other studies with Kis have implied a role for the chromatin reader in transcriptional elongation (Srinivasan et al., 2005, Srinivasan et al., 2008), we demonstrate a selectivity for Kis binding and Kis-mediated elevation of H3K36 methylation as no significant changes were observed at the *shi* promoter in Kis knockdown animals. Further work needs to be done to tease apart the relationship between Kis, H3K36 methylation, and any other HMTs responsible for this modification in the context of *ecr* regulation.

In the field of epigenetics, much work has been done to understand the cross talk between different histone modifications and their cumulative outcome on transcription. For example, H3K36me2 by the HMT dMes-4 was reported to specifically increase H4K16ac, a modification most well known for its role in de-condensation of chromatin structure and maintenance of active gene expression (Bell et al., 2007, Shogren-Knaak et al., 2006, Zhang et al., 2017). We provide evidence for this type of epigenetic cross talk by demonstrating a targeted loss of H3K36 methylation as well as a loss of H4K16 acetylation in a Kis knockdown background. It is therefore plausible that Kis might be affecting transcriptional output by promoting H4K16ac via H3K36me2 cross talk. This may explain why HDAC inhibition with SAHA, seemingly unrelated to histone methylation, is able to restore *ecr-b1* mRNA levels and rescue pruning and memory defects associated with loss of Kis. Consistent with this, SAHA treatment has been previously shown to increase H4K16ac and ultimately increase transcriptional output in cancer cells (Barbetti et al., 2013). Additionally, it may be the case that general HDAC inhibition by SAHA may be universally increasing transcription in a non-specific manner. However, we show here that SAHA treatment alone does not significantly affect either *ecr-b1* mRNA levels or axon pruning. Therefore, the phenotypic effects we observe with SAHA treatment are specific to animals where Kis function is decreased. Additional work is essential to building a complete understanding of the integration of extrinsic and intrinsic cues that control transcriptional regulation.

One interesting observation from our work is that Kis appears to affect pruning of the medial lobes of the MB, while largely leaving the dorsal lobes unaffected. This was the case for both our MARCM analysis as well as our RNAi analysis. It is well established that Ecr-B1 is required for axon pruning of both the dorsal and medial lobes (Lee et al., 2000). Indeed, our data further confirm this, as Ecr-B1 expression does show an effect on the pruning of both the dorsal and medial lobes (Figure 4). Thus, these data suggest that Kis must not be regulating axon pruning solely by promoting the transcription of *ecr-b1* and may also be modulating some other factor that can discriminate between dorsal and medial lobes. Recent work has shown that the neuronal architecture of the MB is quite extensive and forms 15 distinct compartments that tile the MB lobes (Aso et al., 2014a, Aso et al., 2014b). Thus, Kis may only be affecting *ecr-b1* expression and pruning in a subset of the neurons that innervate the medial lobes, or, alternately, may be affecting additional factors besides Ecr-B1 that can discriminate pruning between these two lobes. Determining what these factors may be will require additional work.

In summary, our work has helped to shed light on the epigenetic factors that are involved in the regulated developmental pruning required in Drosophila MB neurons. Determining how axons are properly pruned is fundamental to unraveling the mechanisms that underlie the refinement of neural circuits.

### Limitations of the Study

One limitation of the current study is the method we use to overexpress the Kismet protein, in that the overexpressed RNA can be targeted by our RNAi strategy. Future studies should consider utilizing an overexpression transgene that cannot be targeted by RNAi. A second limitation to the current study is that fact that, although we have identified a protein that differentially affects pruning in the medial vs. the dorsal lobes of the MB, we cannot fully explain how Kismet was able to accomplish this. Future studies should be cognizant that proteins may regulate the pruning of MB lobes differentially.

## Methods

All methods can be found in the accompanying Transparent Methods supplemental file.

## Supplemental Information

Document S1. Transparent Methods, Figures S1–S9, and Tables S1 and S2
