# Supplementary material for: The Drosophila Chromodomain Protein Kismet Activates Steroid Hormone Receptor Transcription to Govern Axon Pruning and Memory In Vivo
Source: iScience. 2019 May 16;16:79–93. doi: 10.1016/j.isci.2019.05.021 (PMC6543131; doi:10.1016/j.isci.2019.05.021)
Supplement: Document S1. Transparent Methods, Figures S1–S9, and Tables S1 and S2 [file mmc1.pdf]

**ISCI, Volume *16***

## Supplemental Information

# The *Drosophila* Chromodomain Protein Kismet Activates Steroid Hormone Receptor Transcription to Govern Axon Pruning and Memory *In Vivo*

**Nina K. Latcheva, Jennifer M. Viveiros, and Daniel R. Marenda**

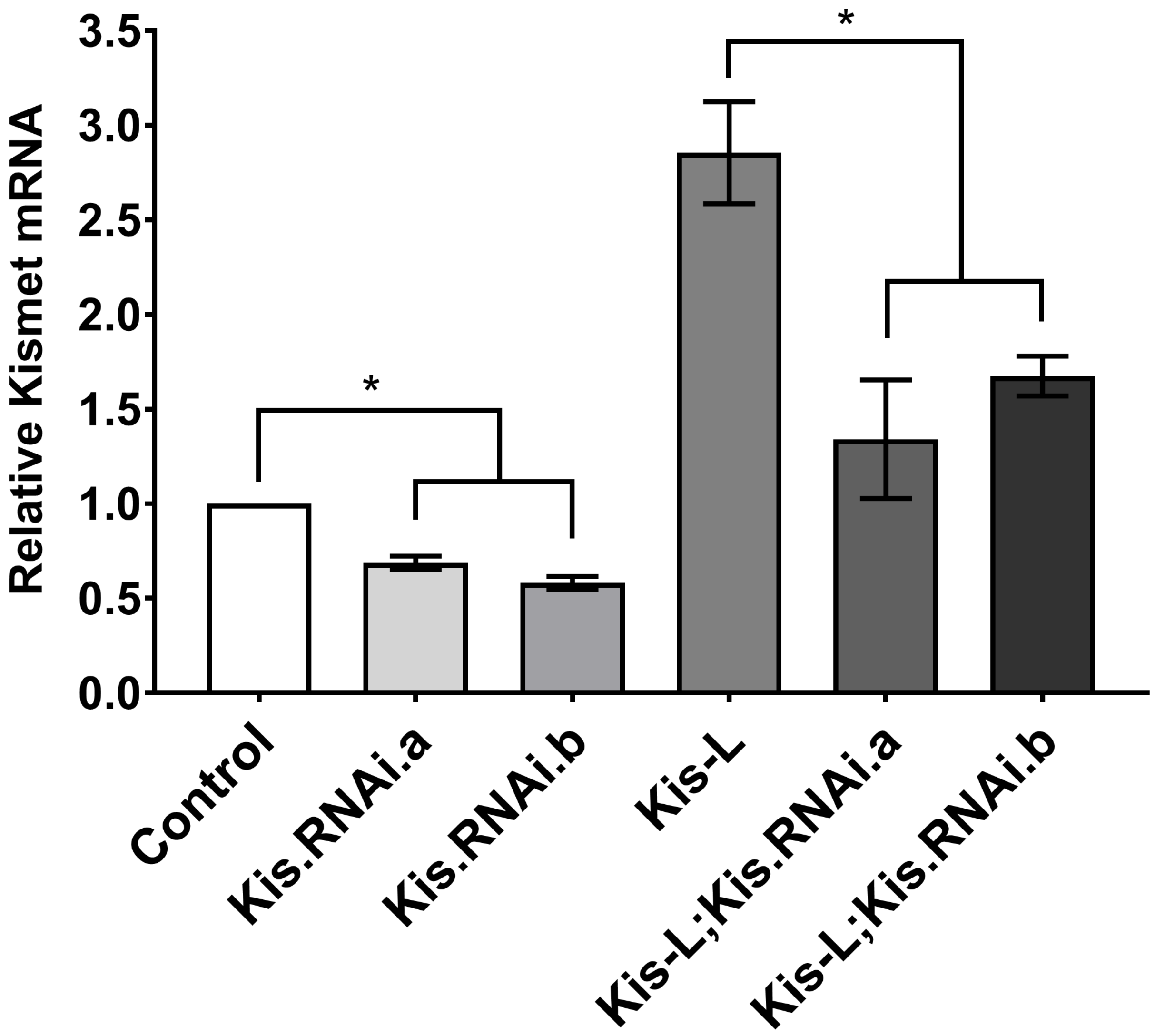


**Figure S1. Exogenous Kis rescues Kis transcript levels in Kis RNAi animals, related to Figure 1.** Quantification of *kis* mRNA levels of control ($w^{1118}$), Kis knockdown (*UAS:kis RNAi.a* and *UAS:kis RNAi.b*), Kis overexpression (*UAS:kis-L*), and Kis rescue (*UAS:kis-L; UAS:kis RNAi.a* and *UAS:kis-L,UAS:kis RNAi.b*) pupal brains analyzed by RT-qPCR (n = 5, 3, 3, 3, 3, 5; 10 brains/ biological replicate). Error bars represent the SEM.

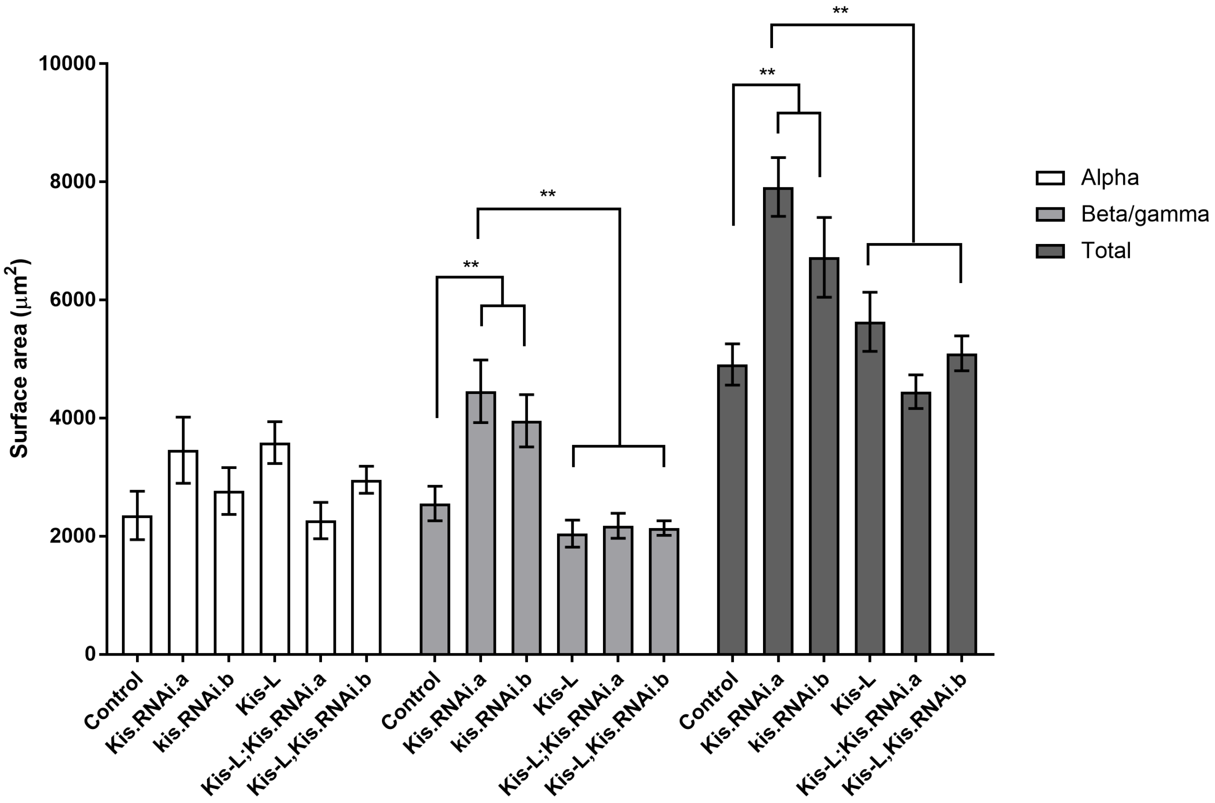


**Figure S2. Kis is required for axon pruning in Kis knockdown pupae, related to Figure 1.** Quantification of dorsal, medial, and total MB lobe surface areas in control ($w^{1118}$), Kis knockdown (*UAS:Kis.RNAi.a* and *UAS:Kis.RNAi.b*), Kis overexpression (*UAS:Kis-L*), and Kis rescue (*UAS:Kis-L; UAS:Kis.RNAi.a* and *UAS:Kis-L,UAS:Kis.RNAi.b*) using the *elav-Gal-4,UAS:mCD8-GFP* driver 18-22 hrs APF (from left to right, n = 9, 9, 8, 8, 9, 10 MBs). Statistical significance is represented by * = $p < 0.05$ and ** = $p < 0.01$. Error bars represent the SEM.

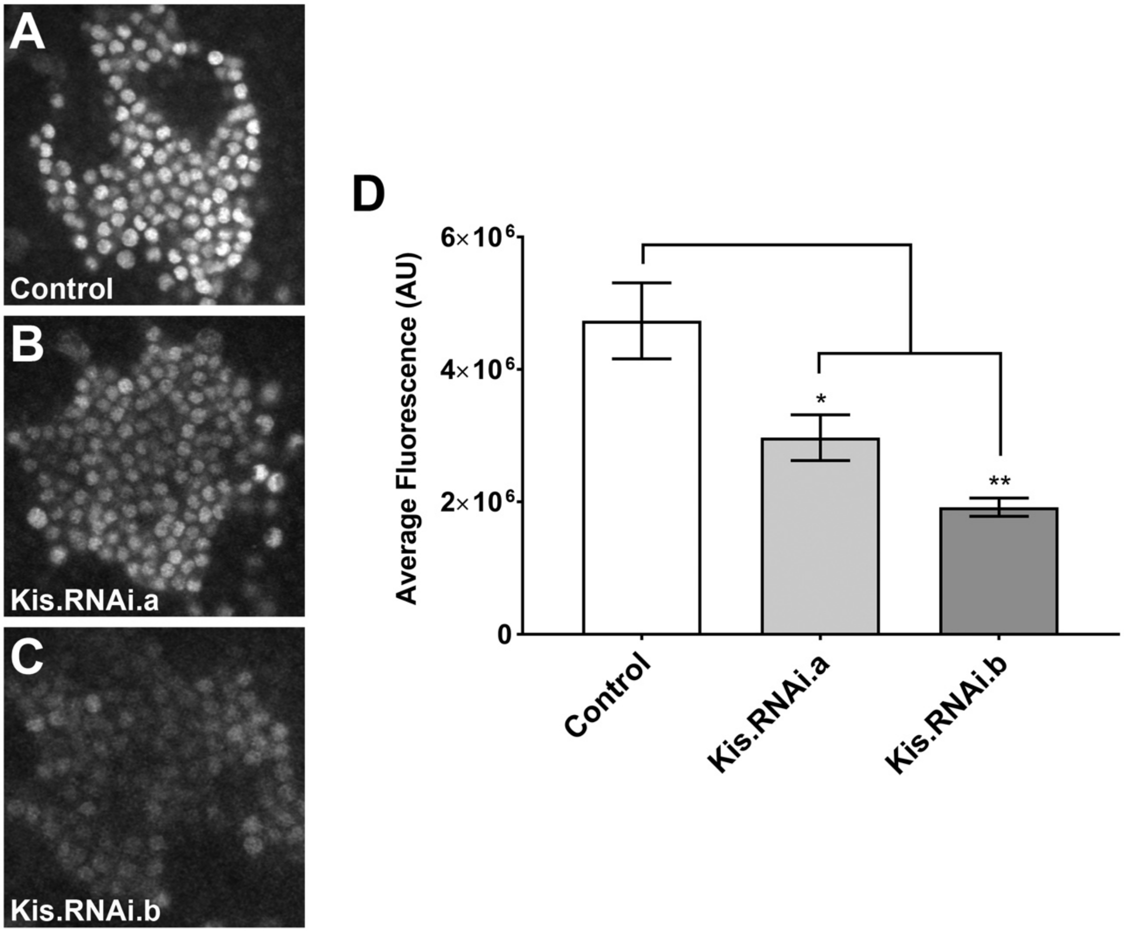


**Figure S3. Kis knockdown decreases EcR-B1 expression in late 3rd instar larva, related to Figure 1.** (A-C) Representative images of EcR-B1 in control (*w*[1118]) and Kis knockdown (*UAS:kis RNAi.a* and *UAS:kis RNAi.b*) late 3rd instar larval Kenyon cells using the *elav-Gal4* driver. (D) Quantification of EcR-B1 within larval Kenyon cells via average fluorescence (n = 5, 6, 4 MBs). Error bars represent the SEM.

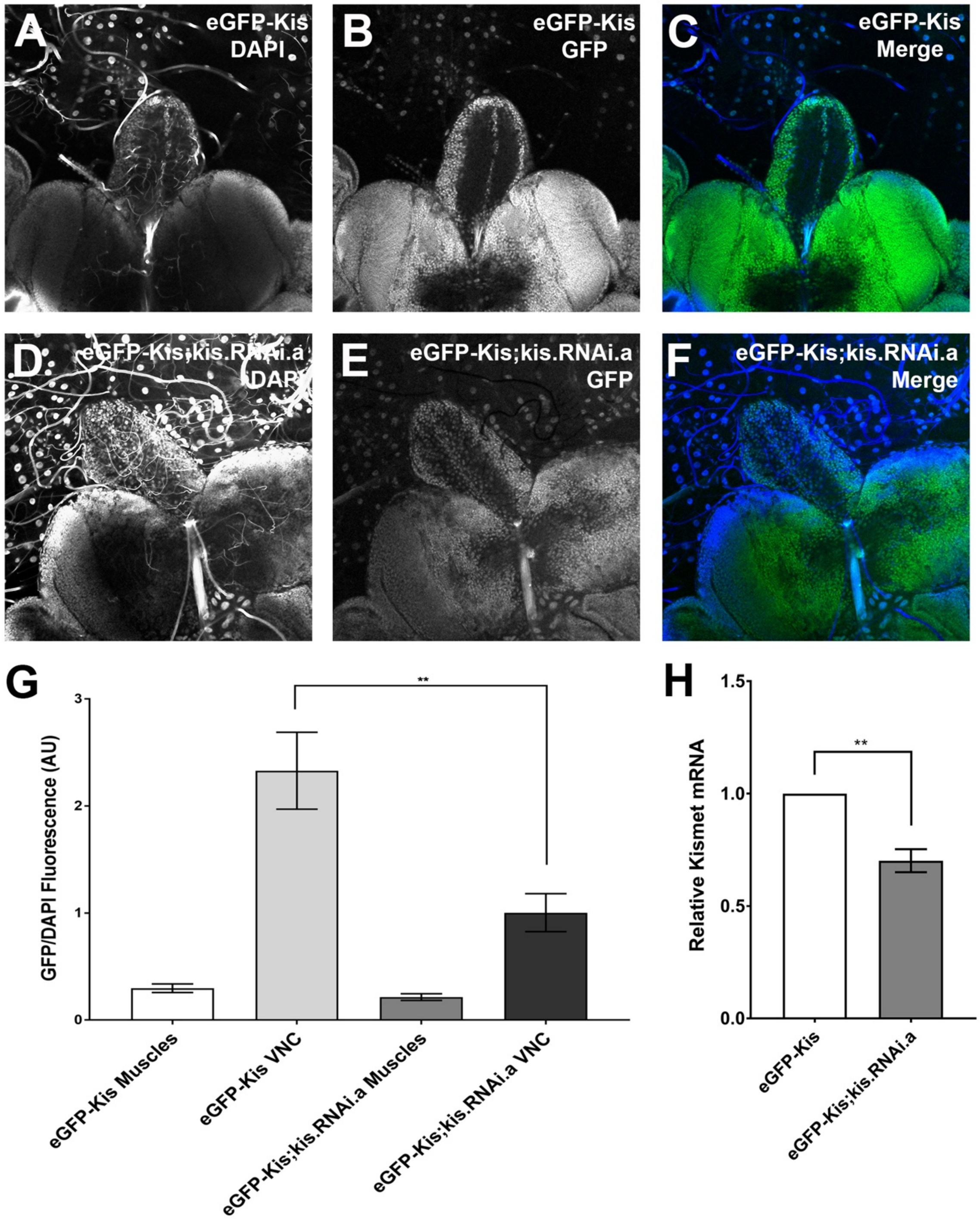


**Figure S4. Kis-eGFP is knocked down by Kis.RNAi.a, related to Figure 2.** (A-F) Representative images of *Kis-eGFP* (control) and *elav-Gal4; Kis-eGFP/Kis-eGFP; kis RNAi.a/+* (Kis knockdown) 3rd instar larval brains stained with DAPI. (G) Quantification of GFP fluorescence intensity compared to that of DAPI ($n = 6$). (H) *kis* mRNA levels of control and Kis knockdown 3rd instar larval brains analyzed by RT-qPCR ($n = 4$, 50 brains/ biological replicate). Statistical significance is represented by ** = $p < 0.01$. Error bars represent the SEM.

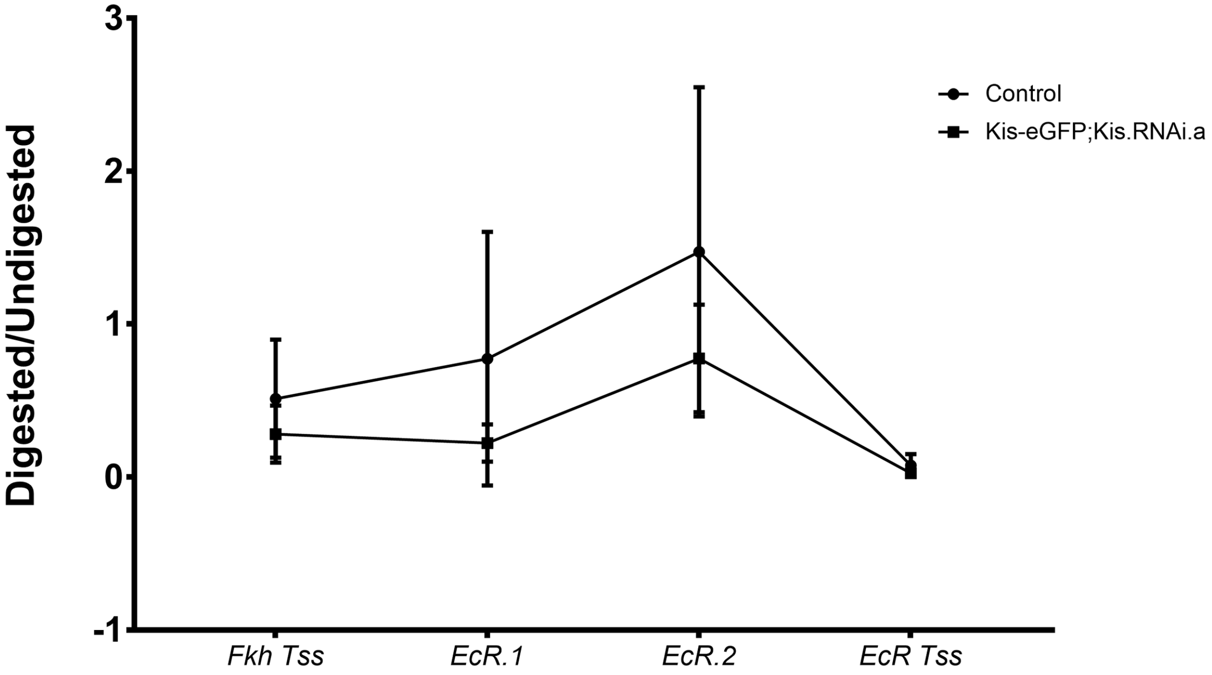


**Figure S5. Kis does not alter DNA protection at *EcR* loci, related to Figure 2.** Quantification of MNase protection assay using *Kis-eGFP* (control) and *elav-Gal4; Kis-eGFP/Kis-eGFP; Kis.RNAi.a/+* (Kis knockdown) 3$^{rd}$ instar larval brains by qPCR at the *ecr* enhancer sites and the *fkh* TSS (n = 6 biological replicates). Error bars represent the SEM.

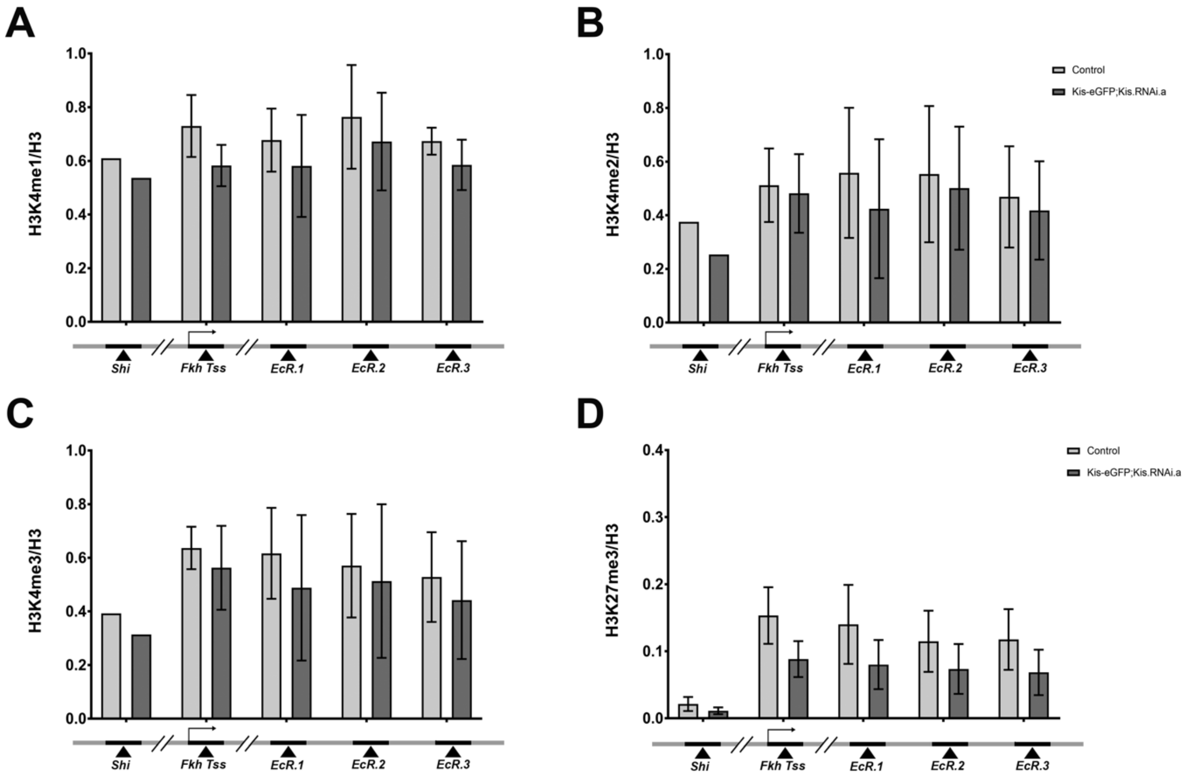


**Figure S6. Kis does not alter H3K4 or H3K27 methylation at *EcR* loci, related to Figure 2.** (A-D) ChIP-qPCR analysis of chromatin isolated from brains of control (*Kis-eGFP*) and Kis knockdown (*elav-Gal4; Kis-eGFP/Kis-eGFP; Kis.RNAi.a/*+) 3rd instar larvae. qPCR analysis of H3K4me1 (n = 2 biological replicates), H3K4me2 (n = 2 biological replicates), H3K4me3 (n = 2 biological replicates), H3K27me3 (n = 4 biological replicates) abundance at the *ecr* enhancer sites, the *fkh* TSS, and the *shi* promoter site, respectively. Error bars represent the SEM.

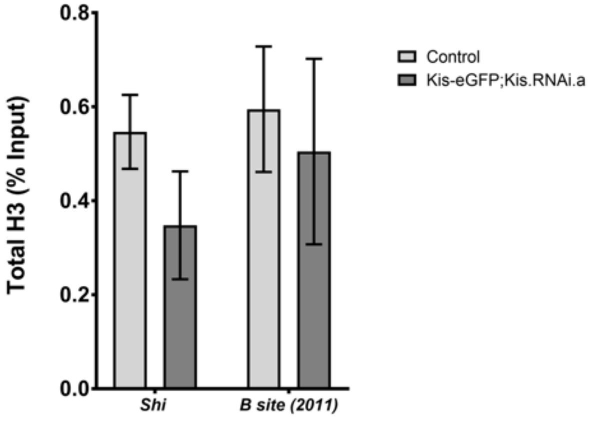


**Figure S7. Kis does not alter total histone 3 at *GMR46E06* locus, related to Figure 3.** Total H3 as a percentage of the input DNA was determined by qPCR at the previously noted genomic loci using *Kis-eGFP* (control) and *elav-Gal4; Kis-eGFP/Kis-eGFP; Kis.RNAi.a*/+ (Kis knockdown) 3$^{rd}$ instar larval brains at the *shi* and the *B site 2011* from Boulanger et al 2011 (Boulanger et al., 2011) (n = 3 biological replicates). Error bars represent the SEM.

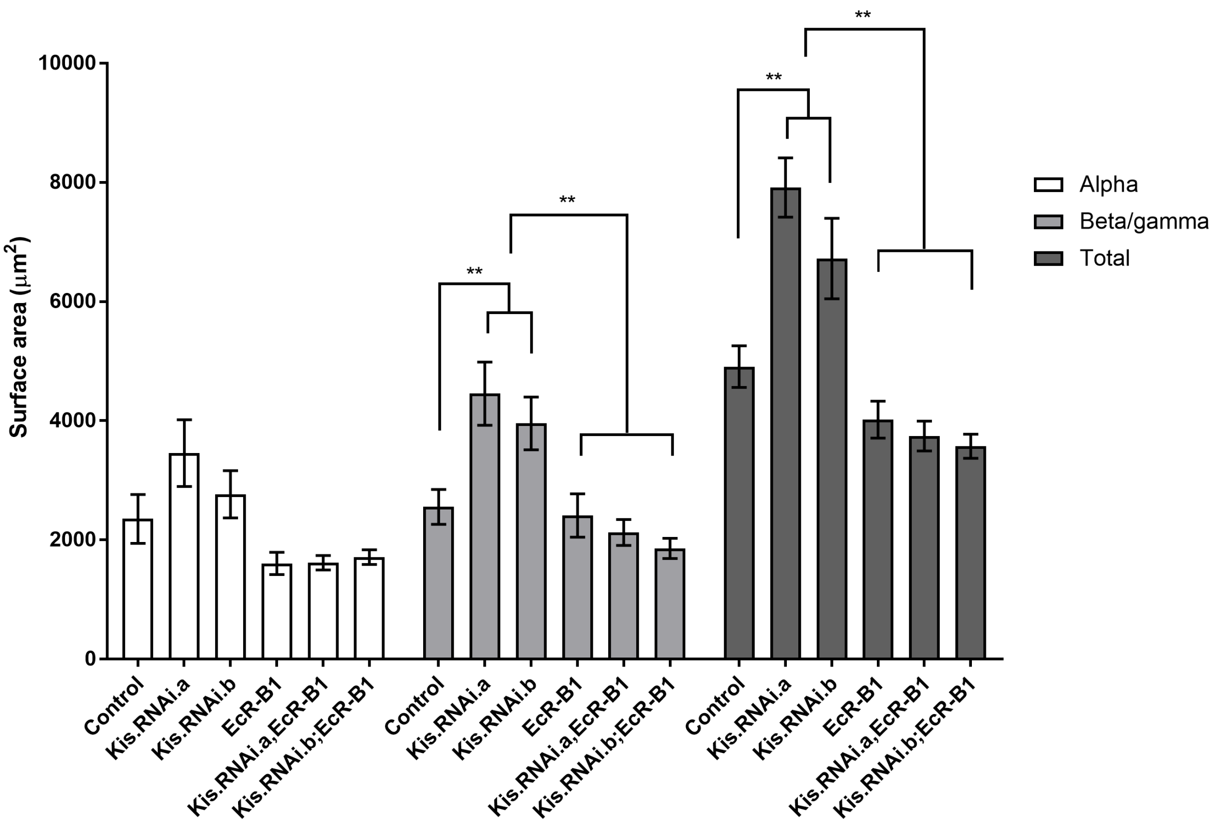


**Figure S8**. **Transgenic EcR-B1 rescues defective axon pruning in Kis knockdown pupae, related to Figure 4.** Quantification of dorsal, medial, and total MB lobe surface areas in control ($w^{1118}$), Kis knockdown (*UAS:Kis.RNAi.a* and *UAS:Kis.RNAi.b*), EcR-B1 overexpression (*UAS:EcR-B1*), and rescue (*UAS:EcR-B1,UAS:Kis.RNAi.a* and *UAS:Kis.RNAi.b; UAS:EcR-B1*) using the *elav-Gal-4,UAS:mCD8-GFP* driver 18-22 hrs APF (n = 9, 9, 8, 7, 10, 11). Statistical significance is represented by ** = $p < 0.01$. Error bars represent the SEM.

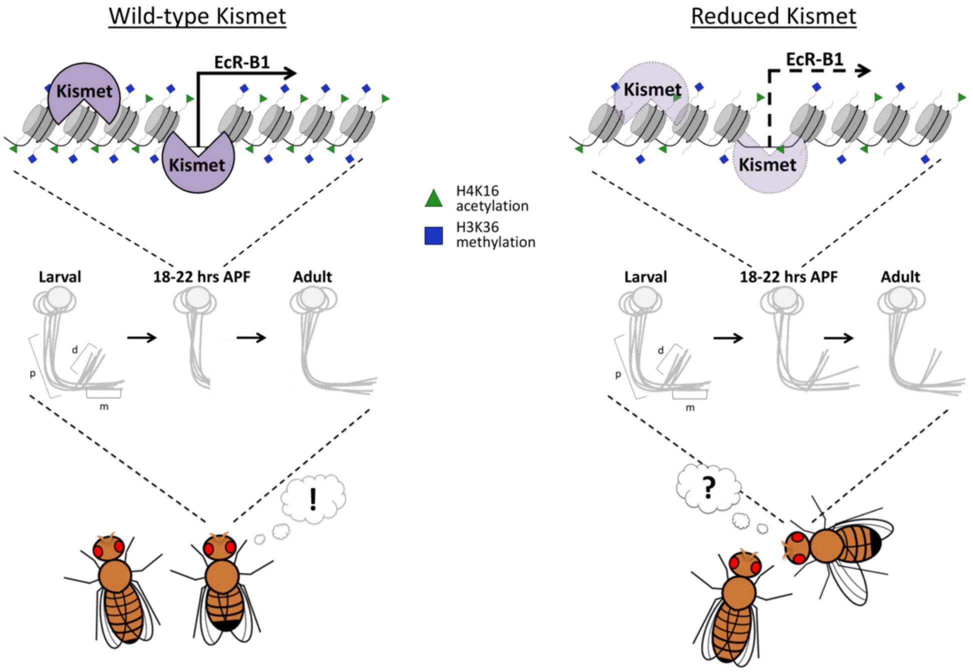


**Figure S9. Model of Kis mediated expression of EcR-B1 necessary for MB pruning and behavior, related to Figures 1-7.** In wild-type flies, Kis promotes the active H3K36 methylation (blue square) and H4K16 acetylation (green triangle) histone marks and binds to cis-regulatory elements of the ecr locus promoting ecr-b1 transcription, which is required for proper developmental MB axon pruning. In Kis knockdown animals, Kis binding to the ecr locus is reduced, thereby decreasing H3K36 methylation and H4K16 acetylation marks leading to decreased ecr-b1 mRNA and EcR-B1 protein. Further, reduction of Kis also leads to defects in MB pruning and immediate recall memory.

**Supplemental Tables**

**Table S1. Quantitative RT-PCR primer sequences, related to Figure 1 and Figure 6**

| Gene of Interest | Forward (5’ to 3’) | Reverse (5’ to 3’) |
|---|---|---|
| *kismet* | GCTCGCATCATACTTCTTTACTG | TCGTGTTTCCACTATTGCTTCC |
| *ecr-b1* | ACT GGC GCA CTA TAT CGA CG | ACATTTTCGCCCGAATCCCT |
| *gal4* | GGATGCTCTTCATGGATTTG | CAACATCATTAGCGTCGGTGAG |
| *rp49* | CTGCTCATGCAGAACCGC | CTGCTCATGCAGAACCGC |

**Table S2. Quantitative PCR primer sequences, related to Figure 2 and Figure 3**

| Gene of Interest | Forward (5’ to 3’) | Reverse (5’ to 3’) |
|---|---|---|
| *fkh TSS* | TCGAGCGGACCAGCAGCTAAA G | TGGGGATTTTTGTTGTCTGCCG |
| *EcR.1* | CGTGGCTAGATCGTTATTAACT G | CGTATTTCGATGGTAGGGTGTC |
| *EcR.2* | GATGTTCGCATACGCGAATACA G | GCAAATTCGCCTCTTTGTTTGTG |
| *EcR.3* | CCGTATCCAACATTCACGTAGA G | TGTATTGCCGAATCGTTGTTGT G |
| *shi pro* | GAAGTGCCAAAGATCAAGTTTG TC | GAGGAAATCCTGTCGCATCTC |

**Transparent Methods**

*Drosophila* stocks and genetics

Unless otherwise noted, all crosses were carried out at 25 °C in a 12:12 light:dark cycle at 60% humidity on standard cornmeal-molasses-agar medium. BL numbers refer to Bloomington Stock Center stock numbers (http://flystocks.bio.indiana.edu/bloomhome.htm). VDRC numbers refer to the Vienna Drosophila Resource Center stock numbers (http://stockcenter.vdrc.at/control/main). To drive the expression of transgenes in *Drosophila*, the Gal4/UAS bipartite system was used as previously described (Brand and Perrimon, 1993). *UAS:Kis.RNAi.a* and *UAS:Kis.RNAi.b* constructs were previously described (Melicharek et al., 2010). The $kis^{LM27}$ allele was generated by EMS mutagenesis, as previously described (Melicharek et al., 2008). *UAS:Kis-L* and *Kis-eGFP* stocks were gifts from J. Tamkun, A. Spradling, respectively (Buszczak et al., 2007; Ghosh et al., 2014). The *201y-Gal4* and *Frt40A* MARCM stocks were gifts from L. Luo (Melicharek et al., 2010). The *ecr* putative enhancer site reporter, *GMR46E06-Gal4*, was obtained as described (Pfeiffer et al., 2011; Pfeiffer et al., 2008). Animals utilized in each assay are listed below.

Assessment of axon pruning (MARCM)
(1) $y^-$,$w^-$,*hs:Flp,UAS:CD8-GFP; tubP-Gal80,Frt40A,201y-Gal4/CyO*
(2) $w^-$; *Frt40A*
(3) $w^-$; $Kis^{LM27}$,*Frt40A/CyO*
(4) $w^-$; *UAS:Kis-L,Frt 40A/CyO*
(5) $w^-$; *UAS:Kis-L,*$Kis^{LM27}$,*Frt40A/CyO*
(6) $w^-$; *Frt40A/CyO; UAS:EcR.B1*
(7) *w-;* $Kis^{LM27}$,*Frt40A/CyO; UAS:EcR.B1*

EcR-B1 staining and assessment of axon pruning (RNAi)
(1) *elav-Gal4,UAS:mCD8-GFP* (BL #5146)
(2) $w^{1118}$ (BL #5905)
(3) $w^-$; *+/+; UAS:Kis.RNAi.a* (VDRC #10762)
(4) $w^-$; *UAS:Kis.RNAi.b* (VDRC #46685)

(5) *w⁻; UAS:Kis-L*
(6) *w⁻; UAS:Kis-L; UAS:Kis.RNAi.a*
(7) *w⁻; UAS:Kis-L,UAS:Kis.RNAi.b*
(8) *w⁻; +/+; UAS:EcR.B1* (BL #6469)
(9) *w⁻; +/+; UAS:EcR.B1/UAS:Kis.RNAi.a*
(10) *w⁻; UAS:Kis.RNAi.b; UAS:EcR.B1*

*ecr-b1* mRNA quantification
(1) *elav-Gal4* (BL #458)
(2) $w^{1118}$
(3) *w⁻; +/+; UAS:Kis.RNAi.a*
(4) *w⁻; UAS:Kis.RNAi.b*
(5) *w⁻; UAS:Kis-L*
(6) *w⁻; UAS:Kis-L; UAS:Kis.RNAi.a*
(7) *w⁻; UAS:Kis-L,UAS:Kis.RNAi.b*

ChIP-qPCR, kis-eGFP validation, MNase protection
(1) *w⁻; Kis-eGFP*
(2) *elav-Gal4; Kis-eGFP*
(3) *w⁻; Kis-eGFP; UAS:Kis.RNAi.a*

Gal4 staining and *gal4* mRNA quantification
(1) *w⁻; +/+; GMR46E06-Gal4* (BL #48166)
(2) $w^{1118}$
(3) *w⁻; +/+; UAS:Kis.RNAi.a*
(4) *w⁻; UAS:Kis.RNAi.b*
(5) *w-; UAS:mCD8-GFP* (BL #5137)

Behavioral testing
(1) *w⁻,hs:Flp,tubP-Gal80,FRT19A; mCD8-GFP/CyO; ok107-Gal4* (BL #44407)
(2) $w^{1118}$
(3) *w⁻; +/+; UAS:EcR.B1*
(4) *w⁻; +/+; UAS:Kis.RNAi.a*
(5) *w⁻; +/+; UAS:EcR.B1/UAS:Kis.RNAi.a*
(6) Canton S

## Pharmacological Treatment

Pharmacological treatment media was prepared as described (Latcheva et al., 2018). Treated fly media was made using dried instant food (Nutri-Fly Instant, Genesee Scientific) with water containing 1.6% of 10% w/v tegosept (methyl p-hydroxybenzoate in 95% ethanol) and 0.1%

of DMSO vehicle or 10μM SAHA. *Drosophila* were raised on drug containing food for their entire lifespan.

## Immunohistochemistry

Immunohistochemical staining was carried out as previously described (D'Rozario et al., 2016). Unless otherwise noted, dissections were performed on Sylgard-coated plates in phosphate buffer and fixed in 4% paraformaldehyde for 25 minutes. In instances of staining, tissues were washed and permeabilized with 0.5% and then 0.1% TritonX-100 in phosphate buffer (0.5% and 0.1% wash buffers, respectively). Tissue was blocked with 10% normal goat serum (in 0.1% wash buffer) before and after incubation with a primary antibody. Overnight incubations with primary and secondary antibodies were performed. Primary antibodies obtained from the Iowa Developmental Hybridoma Bank include α-EcR-B1 (1:200), α-Gal4 (1:200), and α-FasII (1:200). Fluorescently conjugated goat α-rabbit or goat α-mouse secondary antibodies (1:100, Jackson Immunoresearch Labs). Brains were mounted in Vectashield with DAPI (Vector Laboratories, H-1000) and images were obtained using an Olympus Fluoview 1000 laser scanning confocal microscope. Corrected fluorescence intensity was calculated in ImageJ using the following formula: Intergraded Density of selected area – (selected Area * Mean of background).

## MARCM Analysis

Mosaic analysis clones were generated as previously described (Bornstein et al., 2015; Lee and Luo, 1999; Wu and Luo, 2006). A 60-minute heat shock at 37 °C occurred 24 hours after initial egg laying. For pupal MB assessment, white pre-pupae were marked throughout a 4-hour window, were fixed overnight in 2% paraformaldehyde 18-22 hours APF, and then dissected in

phosphate buffer. Pupal MB lobes were visualized via mCD8-GFP and confocal stacks of the dorsal and medial lobes were obtained. In ImageJ, Z-projections of Max Intensity were generated for each lobe and surface area was measured by outlining the mCD8-GFP positive axon bundles. Total lobe surface area was calculated by adding dorsal and medial surface areas together. For adult MB assessment, adults were aged 5 days following eclosion and brains were dissected and fixed in 2% paraformaldehyde. Quantification of the number of aberrant axonal projections was performed by observing MBs in the 3D conformation in ImageJ of mCD8-GFP positive FasII negative projections outside of the dorsal lobe (Bornstein et al., 2015).

Quantitative RT-PCR

10 pupal (approximately 18 hours APF) heads or 50 3$^{rd}$ instar larval brains were dissected in ice-cold phosphate buffer per condition for each biological replicate. These were immediately transferred to RNA Later (Abion) and stored in –80 °C. Isolation of total RNA was done using phenol:chloroform extraction followed by alcohol precipitation for purification. RNA was stored in DEPC water at –80 °C. An adapted version of iTaq$^{TM}$ Universal SYBR® Green One-Step protocol (Bio-Rad) was utilized and samples were run on Bio-Rad C1000 Thermal Cycler CFX96 Real-Time system. Primers were made to *kis, ecr-b1,* and *gal4* mRNAs (IDT). ΔC(t) values were calculated by subtracting the C(t) value of each primer set from C(t) value of *rp49* housekeeping control. Fold change in expression was calculated from ΔΔC(t) values. Each experiment was performed in triplicate with at least three biological replicates.

Chromatin Immunoprecipitation

350 brains from 3rd instar larvae were isolated in ice-cold phosphate buffer per condition for each biological replicate. Brains were transferred to 1X phosphate buffered saline (PBS, Hyclone) and stored at –80 °C. A modified version of truChIP Tissue Chromatin Shearing Kit with SDS Shearing Buffer protocol (Covaris) was used to shear the DNA. Heads were washed twice with 1X PBS and then fixed in Buffer A with 1% methanol-free formaldehyde for 5 minutes at room temperature. Fixing was stopped with Quenching Buffer E followed by incubation for 5 minutes at room temperature. Tissue was pelleted by centrifugation at 4 °C for 5 minutes. Supernatant was removed, and tissue was washed twice with cold 1X PBS. Wash buffer (WB) was removed and tissue was homogenized for 2-3 minutes in 500μL Lysis Buffer (LB) B. Volume was increased to 1mL with LB B followed by incubation on rocker at 4 °C for 20 minutes with 3 second vortex every 10 minutes. Lysed tissue was pelleted and resuspended in WB C. Tissue was washed on rocker for 10 minutes at 4°C at which time it was pelleted, followed by an additional washing with WB C without incubation. Pelleted lysed and washed tissue, largely consisting of nuclei, was resuspended in Covaris SDS Shearing Buffer D. The aggregate of nuclei was incubated with Buffer D for 10 minutes with occasional vortex prior to transfer to a TC 12X12 tube for shearing. Shearing followed the S- and E-Series Shearing recommendations for 10 minutes. 1mL aliquots were stored at –80 °C.

Sheared DNA was confirmed to be within a target range of 100-600 bp fragments. Chromatin was immunoprecipitated using Magna ChIP™ HiSens kit (Millipore). 50uL of sheared chromatin was incubated with antibody-coated Magna ChIP Protein A/G Magnetic Beads for 3 hours. Antibodies against modifications H3K27me3 (rabbit, ab195477), H3K4me1 (rabbit, ab8895), H3K4me2 (rabbit, ab7766), H3K4me3 (rabbit, ab8580), H3K36me2 (rabbit, ab9049), H3K36me3 (rabbit, ab9050), H4K16ac (rabbit, emd millipore 07-329) were used and compared to

Histone H3 (rabbit, ab1791) and Histone H4 (rabbit, ab10158) antibodies, as appropriate. α-GFP (rabbit, ab290) was used to examine Kis abundance and α-IgG (rabbit, ab171870) was utilized as a background control. After elution, samples were incubated with RNaseA (10mg/mL, ThermoScientific) at 37 °C for 30 minutes followed by an incubation with proteinase K (10mg/mL, Millipore) at 57 °C overnight and then inactivate at 75 °C for 15 minutes the next day. Isolated DNA was purified via QIAquick® PCR Purification Kit (Qiagen) and stored at –20 °C.

MNase Protection Assay

350 brains from 3rd instar larvae were dissected in ice-cold phosphate buffer per condition for each biological replicate. Brains were transferred to 1X PBS, and stored at –80 °C. An MNase protection assay was performed using an adapted protocol from (Berson et al., 2017; Chereji et al., 2016). Tissue was homogenized in 500µL of crosslinking buffer (60mM KCl, 15mM NaCl, 4mM MgCl2, 15mM HEPES pH 7.6, 0.5mM DTT, 0.5% Triton X-100, protease inhibitor (100X), 2% formaldehyde) and incubated at room temperature for 15 minutes. Crosslinking was quenched with 50µL of 2.5M of glycine and incubated at room temperature for 5 minutes. Samples were washed twice in crosslinking buffer and twice in D1 buffer (25% glycerol, 5mM Mg Acetate, 50mM Tris pH 8.0, 0.1mM EDTA, 5mM DTT) and resuspended in 1mL of MNase buffer (60mM KCl, 15mM NaCl, 15mM Tris pH 7.4, 0.5mM DTT, 0.25M sucrose, 1.0mM CaCl2). 10units MNase (70196Y, Affymetrix) was added to sample tubes and incubated at 37 °C for 30 minutes. Reaction was quenched with EDTA (final 12.5mM) and SDS (final 0.5%). Samples were equilibrated with NaCl (final 140mM) and incubated with RNaseA (10mg/mL) at 37 °C and then overnight with

proteinase K (10mg/mL) at 65 °C and then 15 minutes at 75 °C the next day. DNA was purified via QIAquick PCR Purification Kit and stored at –20 °C.

Quantitative PCR

Purified DNA was used to prepare PCR reaction mixes according to DyNAmo Flash SYBR Green qPCR Kit (ThermoFisher Scientific). Samples were run using Bio-Rad C1000 Thermal Cycler CFX96 Real-Time system. Primers were made to the EcR.1, EcR.2, and EcR.3 enhancer sites as well as to the B site (Boulanger et al. 2011) of the *ecr locus* (IDT). Additionally, positive and negative control primers were made to the TSS of *fkh* and the *shi* promoter site, respectively (IDT). For control and RNAi knockdown analysis, values were adjusted for input at each primer set and ΔΔC(t) values were calculated by subtracting the adjusted ΔC(t) value of each primer set from the corresponding ΔC(t) IgG control. Fold change in expression was calculated from ΔΔC(t) values. Each experiment was performed in triplicate with at least three biological replicates.

Behavioral testing

To evaluate learning and memory, the canonical fly courtship behavior was used as a readout in an associative conditioning assay described by Siegel and Hall (Siegel and Hall, 1979). Virgin male flies (0 to 6 hours following eclosion) were collected in individual food vials and aged 5 days. Similarly, virgin female wild-type flies were collected, transferred to collective food vials, and aged 5 days. 24 hours before assessment, virgin wild-type females were mated individually using wild-type males. These flies were subsequently separated from virgin females. This behavioral test was executed in a separate room kept at 25°C and 50% humidity, recorded using a Sony DCR-SR47 Handycam with Carl Zeiss optics, and illuminated from below using a constant

115V white light transilluminator. Genotypes of each male were blinded on the day of the assay and all fly transfers were performed without anesthesia. Aged male flies were transferred to mating chambers (Aktogen) each containing a portioned-off mated female fly. Flies were allowed to acclimate for 2 minutes before the assay. Training was recorded and commenced for 60 minutes. After, the male fly was transferred to a clean mating chamber containing a portioned-off virgin female fly. After a 2-minute acclimation period, the divider was removed, and immediate recall was recorded for 10 minutes. Shams experienced the same manipulations however these aged males were not exposed to any fly during the training portion. Digital video analysis of the time spent courting was performed using iMovie software (Apple). Courtship indices were calculated by total time observed performing courtship behaviors divided by total time assayed.

Statistical analysis

All statistical analyses were performed using GraphPad Prism (v. 7.03). Significance was determined at the 95% confidence interval. Unpaired student's *t*-test was used for all experiments, except pupal pruning analysis (utilized two-way ANOVA test) and the learning portion of the associative conditioning assay (utilized paired student's *t*-test). Statistical significance in figures is represented by * = $p < 0.05$, ** = $p < 0.01$, *** = $p < 0.001$, **** = $p < 0.0001$. Error bars represent the standard error of the mean (SEM).

**Supplemental References**

Berson, A., Sartoris, A., Nativio, R., Van Deerlin, V., Toledo, J.B., Porta, S., Liu, S., Chung, C.Y., Garcia, B.A., Lee, V.M., *et al.* (2017). TDP-43 Promotes Neurodegeneration by Impairing Chromatin Remodeling. Curr Biol *27*, 3579-3590 e3576.

Bornstein, B., Zahavi, E.E., Gelley, S., Zoosman, M., Yaniv, S.P., Fuchs, O., Porat, Z., Perlson, E., and Schuldiner, O. (2015). Developmental Axon Pruning Requires Destabilization of Cell Adhesion by JNK Signaling. Neuron *88*, 926-940.

Brand, A.H., and Perrimon, N. (1993). Targeted gene expression as a means of altering cell fates and generating dominant phenotypes. Development *118*, 401-415.

Buszczak, M., Paterno, S., Lighthouse, D., Bachman, J., Planck, J., Owen, S., Skora, A.D., Nystul, T.G., Ohlstein, B., Allen, A., *et al.* (2007). The carnegie protein trap library: a versatile tool for Drosophila developmental studies. Genetics *175*, 1505-1531.

Chereji, R.V., Kan, T.W., Grudniewska, M.K., Romashchenko, A.V., Berezikov, E., Zhimulev, I.F., Guryev, V., Morozov, A.V., and Moshkin, Y.M. (2016). Genome-wide profiling of nucleosome sensitivity and chromatin accessibility in Drosophila melanogaster. Nucleic Acids Res *44*, 1036-1051.

D'Rozario, M., Zhang, T., Waddell, E.A., Zhang, Y., Sahin, C., Sharoni, M., Hu, T., Nayal, M., Kutty, K., Liebl, F., *et al.* (2016). Type I bHLH Proteins Daughterless and Tcf4 Restrict Neurite Branching and Synapse Formation by Repressing Neurexin in Postmitotic Neurons. Cell reports *15*, 386-397.

Ghosh, R., Vegesna, S., Safi, R., Bao, H., Zhang, B., Marenda, D.R., and Liebl, F.L. (2014). Kismet positively regulates glutamate receptor localization and synaptic transmission at the Drosophila neuromuscular junction. PLoS One *9*, e113494.

Latcheva, N.K., Viveiros, J.M., Waddell, E.A., Nguyen, P.T.T., Liebl, F.L.W., and Marenda, D.R. (2018). Epigenetic crosstalk: Pharmacological inhibition of HDACs can rescue defective synaptic morphology and neurotransmission phenotypes associated with loss of the chromatin reader Kismet. Mol Cell Neurosci *87*, 77-85.

Lee, T., and Luo, L. (1999). Mosaic analysis with a repressible cell marker for studies of gene function in neuronal morphogenesis. Neuron *22*, 451-461.

Melicharek, D., Shah, A., DiStefano, G., Gangemi, A.J., Orapallo, A., Vrailas-Mortimer, A.D., and Marenda, D.R. (2008). Identification of Novel Regulators of Atonal Expression in the Developing Drosophila Retina. Genetics *180*, 2095–2110.

Melicharek, D.J., Ramirez, L.C., Singh, S., Thompson, R., and Marenda, D.R. (2010). Kismet/CHD7 regulates axon morphology, memory and locomotion in a Drosophila model of CHARGE syndrome. Hum Mol Genet *19*, 4253-4264.

Pfeiffer, B.D., Jenett, A., Hammonds, A.S., Ngo, T.T., Misra, S., Murphy, C., Scully, A., Carlson, J.W., Wan, K.H., Laverty, T.R*., et al.* (2011). GAL4 Driver Collection of Rubin Laboratory at Janelia Farm.
Pfeiffer, B.D., Jenett, A., Hammonds, A.S., Ngo, T.T.B., Misra, S., Murphy, C., Scully, A., Carlson, J.W., Wan, K.H., Laverty, T.R*., et al.* (2008). Tools for neuroanatomy and neurogenetics in Drosophila. Proceedings of the National Academy of Sciences of the United States of America *105*, 9715-9720.
Siegel, R.W., and Hall, J.C. (1979). Conditioned responses in courtship behavior of normal and mutant Drosophila. Proc Natl Acad Sci U S A *76*, 3430-3434.
Wu, J.S., and Luo, L. (2006). A protocol for mosaic analysis with a repressible cell marker (MARCM) in Drosophila. Nature protocols *1*, 2583-2589.
